# Identifying optimal PD-1/PD-L1 inhibitors in first-line treatment of patients with advanced squamous non-small cell lung cancer in China: Updated systematic review and network meta-analysis

**DOI:** 10.3389/fphar.2022.910656

**Published:** 2022-09-29

**Authors:** Mingye Zhao, Taihang Shao, Yinan Ren, Caicun Zhou, Wenxi Tang

**Affiliations:** ^1^ Department of Pharmacoeconomics, School of International Pharmaceutical Business, China Pharmaceutical University, Nanjing, China; ^2^ Center for Pharmacoeconomics and Outcomes Research, China Pharmaceutical University, Nanjing, China; ^3^ Shanghai Pulmonary Hospital, Tongji University, Shanghai, China

**Keywords:** squamous non-small cell lung cancer, PD-1/PD-L1 inhibitors, NMA, first-line, non-proportional hazard models

## Abstract

**Objective:** After Gemstone-302 was published in Lancet in January 2022, seven PD-(L)1 inhibitors launched or about to be launched in China, but there are no head-to-head RCTs reporting the comparative efficacy for squamous non-small cell lung cancer (sq-NSCLC). Therefore, we aimed to indirectly compare the efficacy of these treatments to provide evidence for clinical decision and Chinese national reimbursement drug listing.

**Methods:** We collected phase III clinical trials targeted on stage IIIB–IV patients for first-line immunotherapy of sq-NSCLC by systematically searching databases. Relative effects of competing treatments were assessed by Bayesian network meta-analysis and non-parametric restricted mean survival time (RMST) model. Hazard ratio (HR), severe adverse events (SAEs, grade 3–5), progression-free survival (PFS) and overall survival (OS) years were the outcomes. Subgroup analysis was done according to PD-(L)1 expression, smoking, gender, Eastern Cooperative Oncology Group performance status, age and disease stage. Sensitivity analysis using the range of parameters distribution as well as different comparison methods was performed to test the robustness of the results.

**Results:** A total of 7 clinical trials with 2,640 patients were included. For OS, the efficiency (HR, 95%CI) ranks from high to low were sugemalimab (0.48, 0.32–0.73), camrelizumab (0.55, 0.40–0.76), sintilimab (0.56, 0.35–0.90), pembrolizumab (0.71, 0.58–0.87) and atezolizumab (0.88, 0.73–1.05). For PFS, the efficiency ranks from high to low were sugemalimab (0.33, 0.24–0.45), camrelizumab (0.37, 0.30–0.46), tislelizumab (0.53, 0.36–0.79), sintilimab (0.54, 0.42–0.69), toripalimab (0.56, 0.38–0.83), pembrolizumab (0.57, 0.47–0.70) and atezolizumab (0.71, 0.59–0.85). Proportional hazard models and non-proportional hazard models showed consistent efficiency ranks. When extrapolated to long-term survival benefit, under non-proportional hazard ratio, sugemalimab achieved the highest PFS benefit (lifeyears, LYs) in 2 years (1.323), with camrelizumab (1.320), sintilimab (1.243), tislelizumab (1.189), pembrolizumab (0.990) and atezolizumab (0.947) ranking in order; Camrelizumab achieved the highest OS benefit (LYs) in 10 years (2.723), with atezolizumab (2.445) and pembrolizumab (2.397) ranking in order. RMST model showed similar results. In terms of safety, PD-(L)1 inhibitors increased the incidence of SAEs when combined with chemotherapy, sugemalimab and camrelizumab was the safest drugs.

**Conclusion:** Sugemalimab is superior both in HR and long-term survival benefit for Chinese patients with advanced sq-NSCLC.

## Introduction

According to the International Agency for Research on Cancer, approximately 19.3 million new cancer cases and nearly 10 million cancer-related deaths occurred in 2020. Lung cancer amounted to 11.4% of the new cancer cases, ranking second only after breast cancer (11.7%). It also contributed to 18% of new cancer-related deaths, ranking first among all cancers. (Sung et al., 2021). Non-small cell lung cancer (NSCLC) accounts for 80%–85% of all lung cancers, (Duma et al., 2019; Leighl 2012), of which, nearly one-third of patients are diagnosed with the squamous histological subtype. (Shi et al., 2021). According to statistical data from China National Cancer Center, both the incidence and mortality rates of lung cancer in China ranked first among all malignant tumors in 2014, with 781,000 new cases and 626,000 deaths. Platinum, gemcitabine, pemetrexed, and paclitaxel are all recommended by the National Comprehensive Cancer Network (NCCN) 2021 guidelines as first-line chemotherapeutic drugs for the treatment of advanced NSCLC. (NCCN Guidelines Version 7.2021-Non-Small Cell Lung Cancer National Comprehensive Cancer Network, 2021) Despite the availability of these therapeutic regimens, patients with advanced NSCLC still have low survival rates. A 2016 study in the United States showed that the 60-months overall survival (OS) rate of stage IB patients was 68%, while the OS rate of stage IVA–IVB patients was only 0%–10%. (Goldstraw et al., 2016). The median progression-free survival (PFS) of patients with stage IIIB–IV NSCLC was 3–8 months, (Galetta et al., 2015; Scagliotti et al., 2008; Scagliotti et al., 2009; Schiller et al., 2002), and the median OS was 7–17 months. (Cardenal et al., 1999; Paz-Ares et al., 2013; Scagliotti et al., 2008; Scagliotti et al., 2009; Schiller et al., 2002).

Programmed death-1 (PD-1) and programmed death-ligand 1 (PD-L1) immune-checkpoint inhibitors have emerged in recent years as a breakthrough in the treatment of NSCLC. PD-L1 is expressed in normal tissues but overexpresses in a variety of tumors. In NSCLC, its expression rate in tumors is up to 35%–95%. (Horita et al., 2017). Cytotoxic agents can exhibit positive immunomodulatory effects by releasing high levels of tumor antigens and reinstating immunosurveillance, and activation of immune cells increases the expression of co-inhibitory PD-(L)1, and immune-checkpoint inhibitors restore or even enhance the ability of immune cells to kill tumor cells by blocking co-inhibitory PD-(L)1 expression. (Postow et al., 2015). Thus, immunotherapy combined with chemotherapy has a potential to improve patient outcomes. Anti-PD-L1 fully human monoclonal antibodies can block PD-L1 and T cells, the interaction between PD-1 and CD80 on immune cells exerts an anti-tumor effect by eliminating the immunosuppressive effect of PD-L1 on cytotoxic T cells. Improvements in outcomes in patients receiving combination therapy may be caused by induction of immunogenic cell death by platinum-based chemotherapy, resulting in the down-regulation of PD-L1 and PD-L2, reducing the number of myeloid suppressor cells, enhancing antigen cross-presentation by dendritic cells, and reducing regulatory T-cell activity. (Paz-Ares et al., 2020). As a human immunoglobulin G4 monoclonal antibody, PD-1 immune-checkpoint inhibitors can specifically bind to PD-1 molecules on the surface of T cells, blocking its interaction with PD-L1/2 and PD-1 pathway-mediated immunosuppressive responses, including anti-tumor immune responses, thereby achieving the purpose of treating tumors (Jotte et al., 2020).

The PD-(L)1 inhibitors recommended by NCCN guidelines for first-line treatment of NSCLC include nivolumab, pembrolizumab, and atezolizumab. (NCCN Guidelines Version 7.2021-Non-Small Cell Lung Cancer, National Comprehensive Cancer Network, 2021) Pembrolizumab were approved in China in 2019, atezolizumab is likely to be approved in the near future, for first-line treatment of squamous Non-small cell lung cancer (sq-NSCLC). Camrelizumab, sintilimab, tislelizumab and sugemalimab, which are manufactured in China, were also approved in 2021. According to the Center for Drug Evaluation, toripalimab will enter the market by the 2022. The indications of sintilimab and tislelizumab, namely, advanced sq-NSCLC, have also been successfully listed in the new round of national health insurance negotiations in November 2021.

The market of PD-(L)1 inhibitors has grown in China over just a few years. Given the highly overlapping treatment areas of these drugs, whether they exhibit similar clinical value has not been fully addressed. A direct comparison of these drugs in the clinic has not been performed in treating sq-NSCLC, and little is known about their differences in terms of clinical efficacy and survival benefits. Therefore, this study aims to indirectly compare and rank the benefits of the seven PD-1/PD-L1 inhibitors that are available on the market or that will enter the market soon in China. The results of this study may provide evidence for solving challenges in clinical decision-making and national health insurance drug catalogue.

## Materials and methods

### Protocol

Our systematic review protocol was drafted using the Preferred Reporting Items for Systematic reviews and Meta-analyses for Protocols (PRISMA-P) guidance. (Shamseer et al., 2015). PRISMA checklist is provided in the online Supplementary Appendix S2. The protocol was revised based on feedback from various stakeholders, including clinical specialists and healthcare professionals. The final protocol was registered with the PROSPERO registry (CRD42021288638) and is presented in the online Supplementary Appendix S3.

### Eligibility criteria

The study populations were ≥18 years of age with stage IIIB to IV sq-NSCLC, PD-L1 expression level was unlimited. The interventions were PD-(L)1 inhibitors that were already in or about to enter the market in China as first-line therapeutic regimens of sq-NSCLC, whose phase III clinical trials were completed and data were available. The intervention group received PD-(L)1 inhibitor combined with chemotherapy, and chemotherapy was limited to pemetrexed plus platinum and paclitaxel/gemcitabine plus platinum, which were approved in China for first-line treatment of advanced sq-NSCLC. The control group received chemotherapy only.

Overall survival (OS), progression-free survival (PFS), and severe adverse events (SAEs, grade 3–5) were the outcome indicators. The corresponding hazard ratio (HR) or odd ratio (OR) and related 95% confidence interval (CI) should also be reported. If a study did not report at least the HR of PFS or OS, it would be excluded.

The studies included were limited to phase III randomized controlled clinical trials. In cases of different published studies or conference abstracts of the same clinical trial, we selected the latest and the most comprehensive version. For the interactions with multiple clinical trials, we selected the trials meeting the limited standards and having similar experimental designs.

### Information sources and literature search

As of April 2022, we systematically searched the PubMed (https://pubmed.ncbi.nlm.nih.gov), Embase (https://www.embase.com), and ClinicalTrials.Gov (https://clinicaltrials.gov) to retrieve clinical trials and published studies of associated drugs. We also searched abstracts in European Society for Medical Oncology, American Society of Clinical Oncology, and World Conference on Lung Cancer. There was no limit for the study period, and the language was limited to Chinese or English. Search strategies are shown in Supplementary Method S1 in the online Supplementary Appendix S1.

### Data extraction and extrapolation

The detailed data of clinical trials were extracted, including experimental design, patient baseline characteristics (including trial NCT number, age, gender, country, stage, ECOG score, smoking status, tumor histological type, and PD-L1 expression status), interventions (medication administration and dosage) and outcome indicators. The efficacy outcomes were OS and PFS, the safety outcome was any SAEs.

### Risk of bias assessment

We assessed the risk of bias of individual trials using the RevManager (version 5.3). The overall bias of a trial was assessed from 7 domains: randomization sequence generation, allocation concealment, blinding of participants and personnel, blinding of outcome assessment, incomplete outcome data, selective reporting and other bias. Judgments were made independently by 2 investigators. Disagreements were resolved by discussion. Risk of bias assessment was incorporated into our interpretation of results.

### Statistical analysis

We used GetData Graph Digitizer (version 2.26) to extract survival data from PFS and OS Kaplan-Meier curves. Guyot’s method was used to reconstruct individual patient data and the survival data were then fitted. (Guyot et al., 2012). This is the most accurate data reproduction method currently known for cases in which individual patient data are not available. (EVIDENCE et al. NICE, 2020). Through visual inspection, the reconstructed curves were consistent with the original curves.

To determine the life years of each treatment regimen, chemotherapy in the Keynote407 (Paz-Ares et al., 2020) with the most mature data (maturity of OS and PFS Kaplan-Meier (KM) curves are 85% and 93%, respectively) was selected as the standard regimen. Then, with chemotherapy as the anchor, the survival rate of each treatment was calculated by the HRs obtained from network meta-analysis (NMA). The latest NICE guidelines stated that it was not enough to consider standard parametric models when reconstructing survival curves (Rutherford et al., 2020). Thus, in addition to using the standard distribution model to fit the survival curve, we also considered fractional polynomial models (FP, including first-order and second-order) (Jansen 2011), RP (Royston-Parmar) model (Rutherford et al., 2020), and RCS (Restricted cubic spline) model (Rutherford et al., 2020). Considering the significant plateau effect brought about by the combination therapy, natural mortality was added to the plateau phase when the survival curve was reconstructed in this study, which were extracted from China’s 6^th^ National Census. (China’s 2010 Population Census, National Bureau of Statistics, 2010).

As primary analysis for OS and PFS, we estimated time-varying HRs by Bayesian parametric survival NMA and compared expected survival curves across treatments. Log cumulative hazards plots indicated proportional hazard ratio were not exist in our study. More details are presented in Supplementary Figure S2. We fit a series of first-order fractional polynomial models with power parameters −2, −1, −0.5, 0.5, 1, 2, and 3, the Akaike information criterion (AIC) was used to assess model fit and choose the best fit first-order models. (Wiksten et al., 2020). Then, non-parametric restricted mean survival time (RMST) model was used to test the short-term survival benefit of the combination regimen compared to chemotherapy, simulation time of RMST model was the shortest of the longest follow-up time of all treatment regimens (Petit et al., 2019).

As secondary analysis, due to lack of OS and PFS curves (PFS: toripalimab; OS: sintilimab (more than 75% survived at the cut-off point of KM curve, resulted in overfitting to tail data as illustrated by Supplementary Figure S3), toripalimab, sugemalimab and tislelizumab), time-invariant HRs Cox proportional hazards (Cox-PH) model between treatment arms from individual trials were analyzed to estimate the overall HRs. Furthermore, subgroup analysis according to PD-(L)1 expression (<1%/1%–50%/>50%), smoking (current or former smoker/non-smoker), gender (male/female), Eastern Cooperative Oncology Group performance status (ECOG, 0/1), age (<65/≥65) and disease stage (Ⅲb/Ⅳ) was performed for both PFS and OS using Cox-PH model.

We calculated life-years (LYs) for each treatment within a certain time to compare the effectiveness of all treatment. We also performed sensitivity analysis using the range of HR-related parameters to test the robustness of the results assuming that the parameters followed a uniform distribution. For SAEs, the number of events in individual trial arms was analyzed to estimate the overall ORs between treatments.

For primary analysis, we used the ggmcmc and R2jags packages in R, with 3 parallel Markov chains consisting of 100,000 samples after a 10,000-sample burn-in. For secondary analysis, Bayesian models estimated treatment effects *via* Markov chain Monte Carlo algorithms. (Dias 2018) We used the gemtc package (gemtc: network meta-analysis using bayesian methods. R package, version 0.8-4. Updated 10 August 2020Van Valkenhoef G, Kuiper J, 2021) in R, version 4.1.0 (R:a language and environment for statistical computing.Published 2019R Foundation for Statistical Computing, 2021) with 4 parallel Markov chains consisting of 50,000 samples after a 10,000 sample burn-in. Convergence of Markov chains was checked by trace plots and Gelman Rubin diagnostic statistics. (BROOKS and Gelman; Gelman1996 BROOKS and Gelman; Gelman 1996). As all comparisons were examined in only 1 trial, there was no sources of inconsistency in our study. The significance level was α = 0.05 for statistical tests.

## Results

### Literature search and study characteristics

**FIGURE 1:**
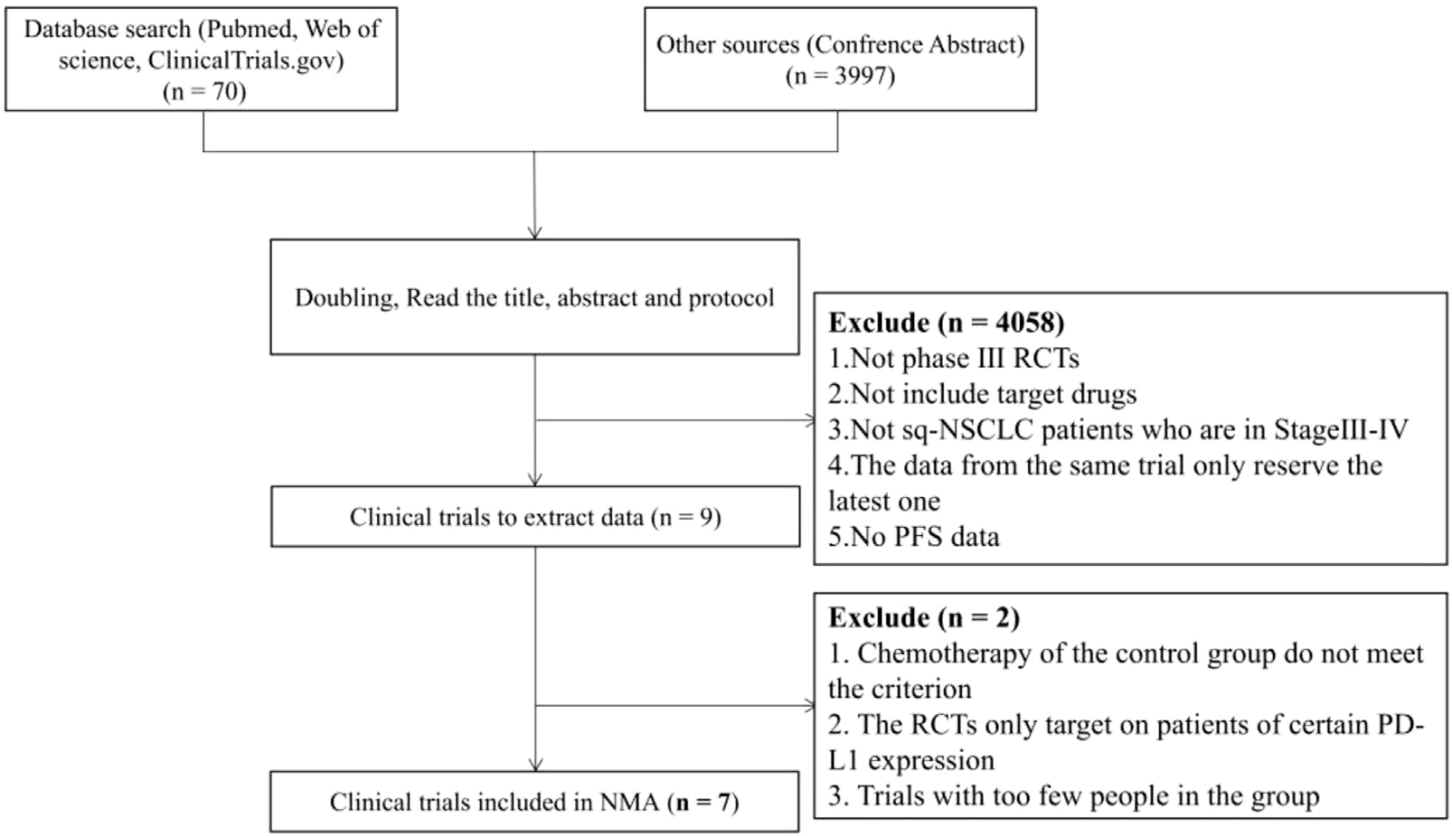
Flow chart of literature search.

A total of 4,067 unique study records were identified, including 59 publication citations, 11 trial regulatory records and 3,997 conference abstracts. After removing duplicates following the preliminary inclusion/exclusion criteria, 9 PD-1/PD-L1 inhibitors and 22 clinical trials were retained. Full-text screening was done for these records. After comparison of the experimental designs and screening for the reporting of outcome indicators, 7 PD-(L)1 inhibitors with 7 clinical trials included 2,640 patients were finally included. The flow chart of the literature search is shown in Figure 1.

**FIGURE 2:**
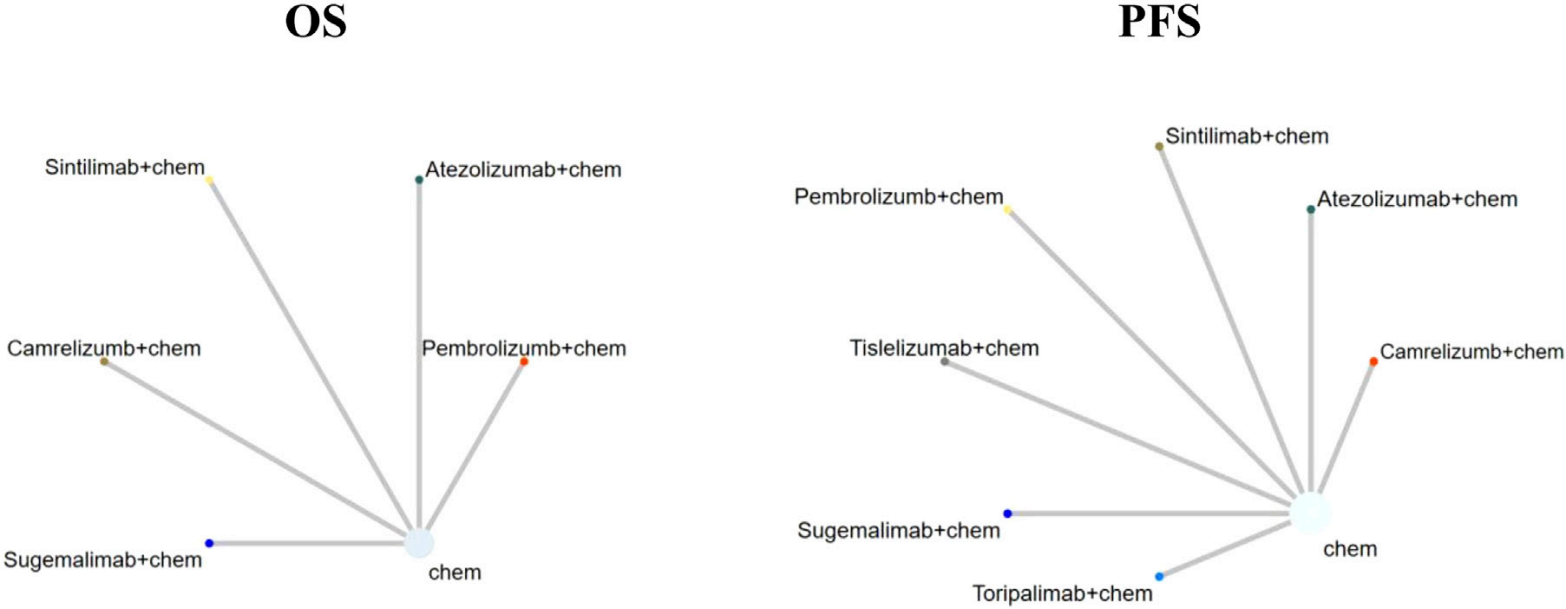
Network plot (Comparisons on overall survival (OS) and progression-free survival (PFS). Each circular node represented a type of treatment. Each line represented a type of head-to-head comparison. The size of the nodes and thickness of lines were weighted according to the number of studies evaluating each treatment and direct comparison, respectively; chem: chemotherapy).

Key information of the included trials was listed in Table 1, patient baseline characteristics are available in the online Supplementary Appendix S1 Supplementary Table S1. Among the 7 clinical trials, 5 trials focused on stage IV patients and 2 trials focused on stage ⅢB-Ⅳ patients (percents of stage IV patients exceeds 75%), 3 clinical trial (Rational307 (Wang et al., 2021), Genstone302 (Zhou et al., 2022), CHOICE-01 (Zhou et al., 2021)) did not report the OS curve, furthermore, CHOICE-01 (Zhou et al., 2021) did not report the PFS curve and HR for overall survival. The network plot for direct and indirect comparison of all treatments are shown in Figure 2 For all RCT studies, the risk of bias .

**TABLE 1:** Key information of included trials.

Study	Intervention arm	Control arm	Clinical stage	HR (95%CI)		SAE/Total. (%)	
				PFS	OS	Intervention arm	Control arm
Keynote407 (Paz-Ares et al., 2020)	Pembrolizumb + chemotherapy	chemotherapy	Ⅳ	0.57 (0.47–0.69)	0.71 (0.58–0.88)	206/278 (74)	195/290 (70)
Impower 131 (Jotte et al., 2020)	Atezolizumab + chemotherapy	chemotherapy	Ⅳ	0.71 (0.60–0.85)	0.88 (0.73–1.05)	277/334 (83)	235/334 (70)
Orient12 (Zhou et al., 2021)	Sintilimab + chemotherapy	chemotherapy	ⅢB-Ⅳ	0.536 (0.422–0.681)	0.567 (0.353–0.909)	155/179 (87)	148/178 (82)
CAMEL-sq (Zhou et al., 2021)	Camrelizumb + chemotherapy	chemotherapy	Ⅳ	0.37 (0.29–0.47)	0.55 (0.4–0.75)	142/193 (74)	141/196 (72)
Rationale307 (Wang et al., 2021)	Tislelizumab + chemotherapy	chemotherapy	ⅢB-Ⅳ	0.52 (0.37–0.74)	NA	106/120 (88)	98/117 (84)
Gemstone302 (Zhou et al., 2022)	Sugemalimab + chemotherapy	chemotherapy	Ⅳ	0.34 (0.24–0.48)	0.48 (0.31–0.74)	205/320 (64)	98/159 (62)
CHOICE-01 (Wang et al., 2021)	Toripalimab + chemotherapy	chemotherapy	Ⅳ	0.55 (0.38–0.83)	NA*:	NA	NA

HR, hazard ratios; OS, overall survival; PFS, progression-free survival; SAE, severe adverse events.* The HR reported by the study does not subdivide sq and nsq.

### Risk of bias

For all RCT studies, the risk of bias was generally low. Risk of bias assessment graph is presented in online Supplementary Appendix S1; Supplementary Figure S1. Specifically, only blinding of participants and personnel raised bias in Impower 131 (Jotte et al., 2020), CAMEL-sq (Zhou et al., 2021), CHOICE-01 (Zhou et al., 2021) and Rationale 307 (Wang et al., 2021).

### Overall outcomes

RP models were fitted to the OS and PFS Kaplan-Meier survival curves for chemotherapy in the Keynote407 (Paz-Ares et al., 2020) (knot = 1, scale of hazard for OS and knot = 5, scale of hazard for PFS), which demonstrated the best fit for the KM survival data. We didn’t consider standard parametric, FP or RCS models as their poor fitting to the chosen KM curves. More details are provided in Supplementary Table S2 and Supplementary Figure S3 in the Supplementary Appendix S1.

**FIGURE 3:**
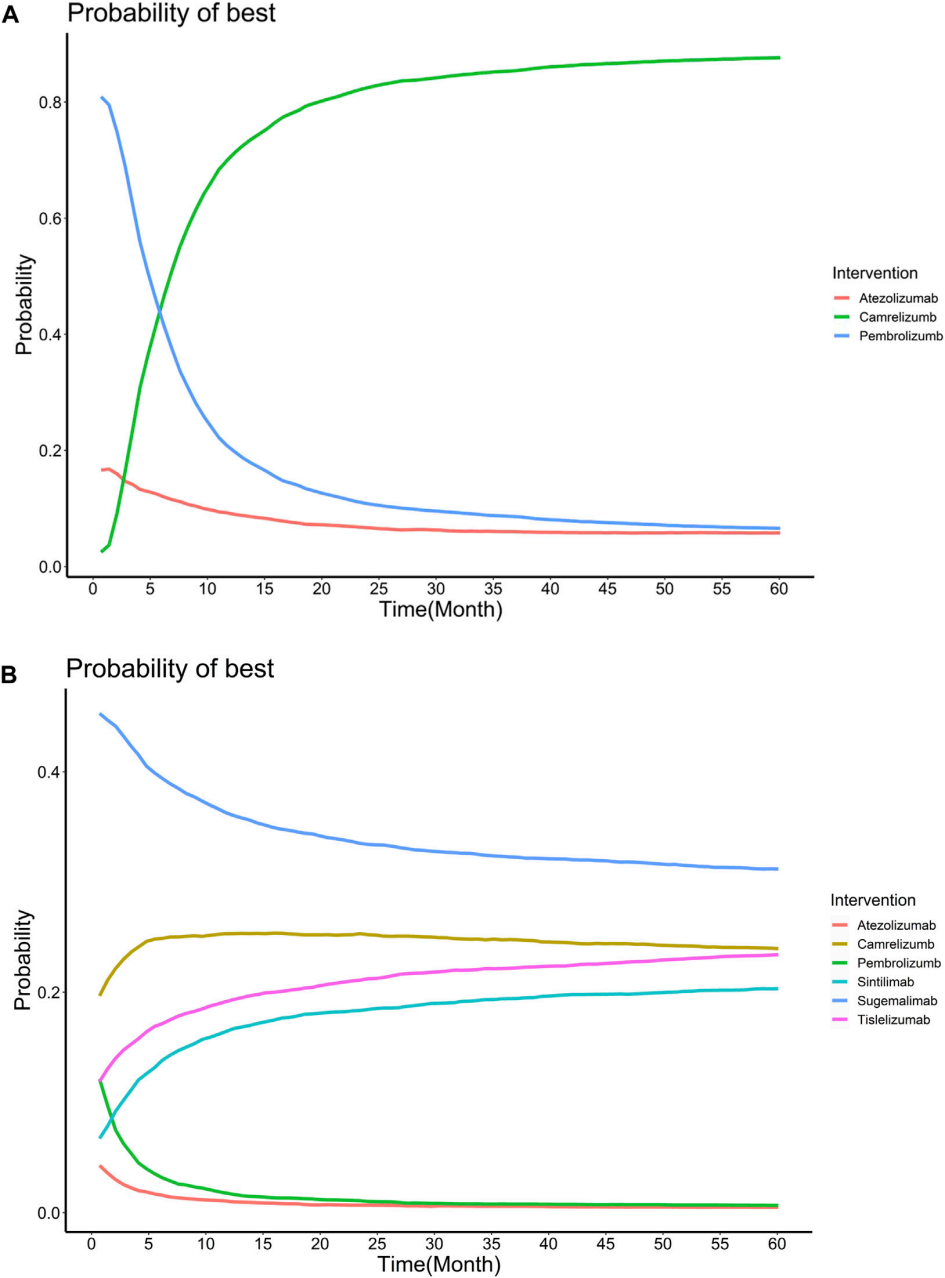
Treatment probabilities of optimal effectiveness over time for overall survival **(A)** and progression-free survival **(B)** for different regimens added to chemotherapy.

Allowing the HR to change over time in the primary analysis. The first-order FP model fitted OS data and PFS data best when power equals to −1 and −0.5, respectively. The OS and PFS curves fitted by all FP models and AIC for each model are provided in Supplementary Figure S4 and Supplementary Table S3. Supplementary Figure S5 shows the expected OS and PFS curves of 60 months for each treatment, which were relied on the estimated time-varying HRs of each treatment relative to chemotherapy and subsequently applied to a parametric reference curve with chemotherapy obtained from the Keynote-407. (Jotte et al., 2020). When follow-up time reached 24 months, sugemalimab achieved the highest PFS benefit (1.323 LYs), with camrelizumab (1.320 LYs), sintilimab (1.243 LYs), tislelizumab (1.189 LYs), pembrolizumab (0.990 LYs) and atezolizumab (0.947 LYs) ranking in order. When extrapolated to 120 months, camrelizumab achieved the highest OS benefit (3.723 LYs), atezolizumab (2.445 LYs) and pembrolizumab (2.397 LYs) ranking in order. More details are concluded in Table 2. Treatment probabilities of optimal effectiveness over time for OS and PFS calculated by FP model are presented in Figure 3, which showed that sugemalimab had the greatest probability of being optimal effectiveness over time for PFS, pembrolizumab and camrelizumab had the greatest probability of being optimal effectiveness before and after 7 months for OS. The HR related parameters are presented in Supplementary Table S4. RMST model showed that compared with chemotherapy monotherapy, sugemalimab gained additional 3.02 months during about 1-year for PFS, followed by camrelizumab (2.72), tislelizumab (1.76), pembrolizumab (1.71), sintilimab (1.53) and atezolizumab (0.90); For OS, pembrolizumab gained additional 1.02 months during about 1-year, followed by camrelizumab (0.91), sintilimab (0.61) and atezolizumab (−0.11), more details are provided in Table 2, related ranking probabilities are presented in Supplementary Figure S6.

**TABLE 2:** Overall survival and progression-free survival estimates for different regimens added to chemotherapy.

Time/year	Pembrolizumb	Atezolizumab	Camrelizumb	Sintilimab	Sugemalimab	Tislelizumab	Toripalimab
Parametric models: Survival years based on FP and Cox-PH models							
PFS: time-varying HRs							
1	0.663	0.640	0.765	0.728	0.775	0.710	—
2	0.990	0.947	1.320	1.243	1.323	1.189	—
PFS:time-invariant HRs							
1	0.568	0.523	0.683	0.622	0.695	0.627	0.617
2	0.820	0.699	1.200	0.981	1.248	1.001	0.967
OS: time-varying HRs							
5	2.054	2.058	2.757	—	—	—	—
10	2.397	2.445	3.723	—	—	—	—
OS: time-invariant HRs							
5	2.112	1.802	2.476	2.419	2.702	--	--
10	2.495	2.031	3.110	2.994	3.597	--	--
Nonparametric model: Aditional survival years (95% CI) based on RMST model							
OS							
0.98	1.02 (0.42–1.62)	−0.11 (0.66–0.44)	0.61 (0.04–1.18)	(0.19-1.65)	—	—	—
PFS							
1.03	1.71 (1.11–2.31)	0.90 (0.37–1.43)	2.72 (1.06–3.39)	1.53 (0.91–2.15)	3.02 (2.11–3.94)	1.76 (0.83-2.70)	—

FP, fractional polynomial; PH, proportional hazards; RMST, restricted mean survival time

**FIGURE 4:**
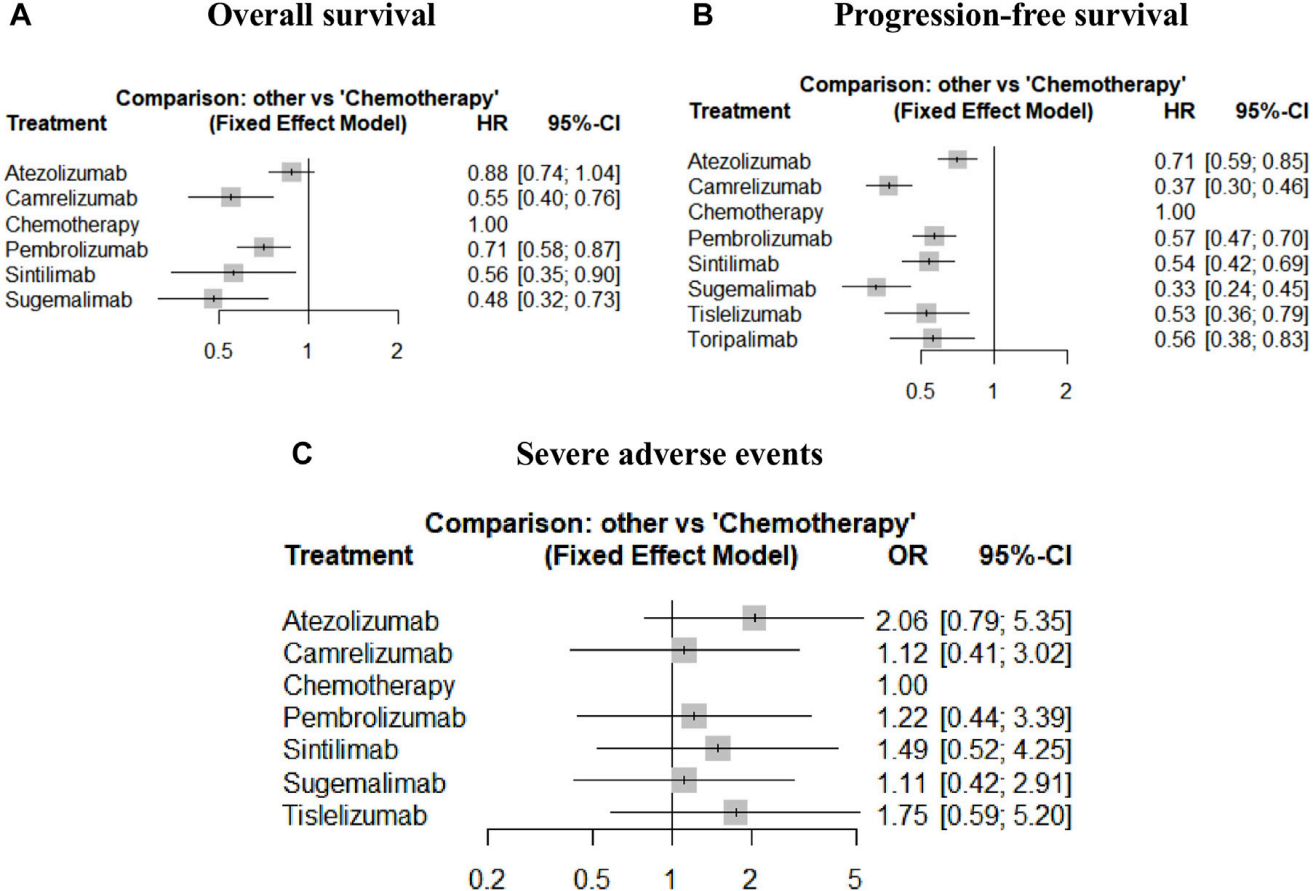
Forest plots of relative effect for overall survival **(A)**, progression-free survival **(B)** and severe adverse events **(C)**.

Assumed HRs were constant over time, the Bayesian network meta-analysis provided consistent treatment rankings for Cox-PH (proportional hazards) model. Ordered from the most to the least effective, treatments with significantly improved OS when combined with chemotherapy included sugemalimab (HR,0.48; 95%CI, 0.32–0.73), camrelizumab (HR,0.55; 95%CI, 0.40–0.76), sintilimab (HR, 0.56; 95%CI, 0.35–0.90), pembrolizumab (HR,0.71; 95%CI, 0.58–0.87). However, no significant improvement was found for atezolizumab (HR, 0.88; 95%CI, 0.74–1.04). For PFS, treatments with significant improvement included sugemalimab (HR, 0.33; 95%CI, 0.24–0.45), camrelizumab (HR, 0.37; 95%CI, 0.30–0.46), tislelizumab (HR, 0.53; 95%CI, 0.36–0.79), sintilimab (HR, 0.54; 95%CI, 0.42–0.69), toripalimab (HR, 0.56; 95%CI, 0.38–0.83), pembrolizumab (HR, 0.57; 95%CI, 0.47–0.70) and atezolizumab (HR, 0.71; 95%CI, 0.59–0.85). Forest plots are provided in Figure 4. Table 3 shows the league tables presenting the overall time invariant HR of PFS and OS for all possible pairwise comparisons between treatments. Life-years for each treatment are concluded in Table 2. Treatment ranking probabilities were provided in Supplemntary Figure S6, which suggested that sugemalimab had the highest probability of being the best treatment regarding OS (probability of 42%) and PFS (probability of 36%).

**TABLE 3:** Relative effect estimates for all possible pairwise treatment comparisons for overall survival, progression-free survival.

Overall survival, hazard ratio (95% CI)							
Pembrolizumb							
1.29 (0.88, 1.90)	Camrelizumb						
1.27 (0.75, 2.13)	0.98 (0.55, 1.75)	Sintilimab					
1.48 (0.93, 2.36)	1.15 (0.67, 1.95)	1.17 (0.62, 2.20)	Sugemalimab				
0.81 (0.62, 1.05)	0.63 (0.43, 0.90)	0.64 (0.38, 1.06)	0.55 (0.35, 0.86)	Atezolizumab			
0.71 (0.58, 0.87)	0.55 (0.40, 0.76)	0.56 (0.35, 0.90)	0.48 (0.32, 0.73)	0.88 (0.74, 1.04)	Chemotherapy		
Progression-free survival, hazard ratio (95% CI)							
Pembrolizumb							
1.54 (1.15, 2.07)	Camrelizumb						
1.08 (0.69, 1.68)	0.70 (0.44, 1.10)	Tislelizumab					
1.06 (0.77, 1.45)	0.69 (0.50, 0.95)	0.98 (0.62, 1.56)	Sintilimab				
1.73 (1.19, 2.51)	1.12 (0.77, 1.64)	1.61 (1.02, 2.82)	1.64 (1.10, 2.43)	Sugemalimab			
0.80 (0.61, 1.06)	0.52 (0.39, 0.69)	0.74 (0.09, 6.02)	0.75 (0.09, 6.16)	0.46 (0.32, 0.67)	Atezolizumab		
1.02 (0.12, 8.4)	0.66 (0.46, 1.04)	0.95 (0.54, 1.66)	0.96 (0.61, 1.53)	0.59 (0.35, 0.98)	1.27 (0.82, 1.96)	Toripalimab	
0.57 (0.47, 0.70)	0.37 (0.30, 0.46)	0.53 (0.36, 0.79)	0.54 (0.42, 0.69)	0.33 (0.24, 0.45)	0.71 (0.59, 0.85)	0.56 (0.38, 0.83)	Chemotherapy
Serious adverse events, odds ratio (95% CI)							
Tislelizumab							
1.58 (0.37, 6.80)	Sugemalimab						
1.18 (0.26, 5.31)	0.74 (0.18, 3.06)	Sintilimab					
1.43 (0.34, 6.09)	0.91 (0.24, 3.47)	1.21 (0.3, 5.07)	Pembrolizumb				
1.57 (0.37, 6.89)	0.99 (0.25, 3.91)	1.33 (0.32, 5.71)	1.1 (0.28, 4.31)	Camrelizumb			
0.85 (0.39, 1.68)	0.54 (0.14, 2.1)	0.72 (0.18, 3.03)	0.59 (0.15, 2.27)	0.54 (0.14, 2.12)	Atezolizumab		
1.75 (0.59, 5.21)	1.11 (0.42, 2.89)	1.49 (0.52, 4.24)	1.22 (0.47, 3.16)	1.12 (0.49, 2.99)	2.06 (0.78, 5.26)	Chemotherapy	

### Safety outcomes

According to the overall ORs compared with chemotherapy, treatments ordered from the safest to the least safe regarding SAEs were sugemalimab (OR, 1.11; 95% CI, 0.42–2.91), camrelizumab (OR, 1.12; 95%CI, 0.41–3.02), pembrolizumab (OR, 1.22; 95%CI 0.44–3.39), sintilimab (OR, 1.49; 95%CI, 0.52–4.25), tislelizumab (OR, 1.75; 95%CI, 0.59–5.20) and atezolizumab (OR, 2.06; 95%CI, 0.79–5.35). More details can be seen in Figure 4 and Table 3. Treatment ranking probabilities suggested that camrelizumb had the highest probability of being the safest (24%) regarding SAEs and sugemalimab was a close second. Atezolizumab had the highest probability of being the least safe treatment (54%). Details for rank probabilities for all possible treatment comparisons are available in Supplemntary Figure S6.

### Subgroup analysis outcomes

For OS, in the subgroups of PD-L1 expression <1% and 1%–50%, camrelizumab was the most effective among the drugs for which subgroup data were available, followed by pembrolizumab, while in the subgroup of PD-L1 expression >50%, atezolizumab and carrelizumab showed similar efficacy, both were better than pembrolizumab. In the subgroup of disease stage Ⅳ patients, sugalimumab was the most effective, followed by camrelizumab. Camrelizumab was the most effective, followed by pembrolizumab for male patients, while pembrolizumab were the optimal choice for female patients. Camrelizumab was the better choice for current or former smoker compared to atezolizumab, while atezolizumab performed better for non-smoker. Camrelizumab and pembrolizumab were the best options for patients with ECOG = 0 and ECOG = 1, respectively.

For PFS, among the drugs for which subgroup data were available, camrelizumab was consistently the most effective drug regardless of PD-L1 expression levels, at the same time, it is worth noting that the effiency of atezolizumab in patients with high PD-L1 expression was significantly improved. Camrelizumab and sugalimumab were the most effective drugs for stage III and IV patients, respectively. In the subgroups male and female patients, camrelizumab and pembrolizumab were the optimal choice, respectively, which were consitent to OS. Sintilimab and tislelizumab were the best options for patients under and over 65 years of age, respectively. Camrelizumab was the better choice for current or former smoker compared to atezolizumab, while tislelizumab performed better for non-smoker. Tislelizumab and pembrolizumab were the best options for patients with ECOG = 1 and ECOG = 0 among the drugs for which subgroup data were available, respectively. Details of subgroup analysis results are presented in Supplementary Figure S7.

## Discussion

This study conducted a comprehensive search for eligible RCTs, critically appraised trial quality, synthesized trial data, and ranked treatments by efficacy and safety shown in randomized clinical trials. We identified 7 eligible trials constructing a network meta-analysis in which all treatments had not been compared in head-to-head trials, which highlighted the importance of our study. Sugemalimab with 1.323 LYs gained in 2 years had the greatest probability of being optimal effectiveness over time for PFS compared with camrelizumab (1.320 LYs), sintilimab (1.243 LYs), tislelizumab (1.189 LYs), pembrolizumab (0.990 LYs) and atezolizumab (0.947 LYs) ranking in order. Camrelizumab achieved the highest OS benefit in 10 years (2.723 LYs), with atezolizumab (2.445 LYs) and pembrolizumab (2.397 LYs) ranking in order. Pembrolizumab and Camrelizumab had the greatest probability of being optimal effectiveness before and after 7 months for OS, respectively. Using nonparametric RMST model, compared with chemotherapy monotherapy, sugemalimab gained additional 3.02 months during about 1-year for PFS, followed by camrelizumab (2.72), tislelizumab (1.76), pembrolizumab (1.71), sintilimab (1.53) and atezolizumab (0.90); For OS, pembrolizumab gained additional 1.02 months during about 1-year, followed by camrelizumab (0.91), sintilimab (0.61) and atezolizumab (-0.11).

Assumed HR being constant over time, the ranks were consistent with primary analysis. All drugs were associated with significantly improved OS and PFS when combined with chemotherapy except atezolizumab. For OS, the efficiency ranks from high to low were sugemalimab (HR, 0.48; 95%CI, 0.32–0.73), camrelizumab (HR, 0.55; 95%CI, 0.40–0.76), sintilimab (HR, 0.56; 95%CI, 0.35–0.90), pembrolizumab (HR, 0.71; 95%CI, 0.58–0.87), atezolizumab (HR, 0.88; 95%CI, 0.73–1.05). For PFS, the efficiency ranks from high to low were sugemalimab (HR, 0.33; 95%CI, 0.24–0.45), camrelizumab (HR, 0.37; 95%CI, 0.30–0.46), tislelizumab (HR, 0.53; 95%CI, 0.36–0.79), sintilimab (HR, 0.54; 95%CI, 0.42–0.69), toripalimab (HR, 0.56; 95%CI, 0.38–0.83), pembrolizumab (HR, 0.57; 95%CI, 0.47–0.70) and atezolizumab (HR, 0.71; 95%CI, 0.59–0.85). In terms of safety, PD-(L)1 inhibitors increased the incidence of SAEs when combined with chemotherapy, camrelizumb and sugemalimab were the safest drugs among the all regimens. But it is worth noting that we did not distinguish the types of adverse reactions or consider grade 1–2 AEs, therefore, our results may have some differences between clinical consensus. For example, the incidence of reactive capillary endothelial proliferation caused by camrelizumb was significantly higher than that of the other immunotherapies.

On the one hand, sugemalimab can bind to PD-L1 on the surface of tumor cells through the Fab fragment, block PD-1 and PD-L1 signaling channels, activate T cells, and enhance the anti-tumor effect of T cells; on the other hand, sugemalimab can bind to the FcγR on the surface of macrophages through the antibody Fc segment, activate antibody-dependent cell-mediated phagocytosis (ADCP), and induce macrophages to further kill tumors. Tumor-associated macrophages are divided into M1 and M2 phenotypes, and the latter is denser in advanced patients. This transition may affect the phagocytic function of macrophages, thereby affecting the prognosis of patients. Sugemalimab can promote the transformation of macrophages from M2 type to the more favorable M1 type and restore the role of macrophages in killing tumor cells. (Dahan R et al., 2015; Chen et al., 2012; Viswanathan et al., 2020). The above information may explain the superior activity of sugemalimab for Chinese patients with advanced sq-NSCLC.

After searching Pubmed for published indirect comparison studies with the keyword “Immune target inhibitor, squamous, non-small cell lung cancer,” a total of 8 studies that indirectly compared PD-(L)1 inhibitors in the treatment of advanced sq-NSCLC were found. He (He et al., 2021) compared the therapeutic efficacy for sq-NSCLC (PD-L1≥50%) of atezolizumab combined with chemotherapy, pembrolizumab combined with chemotherapy and chemotherapy, and found that the combined therapy had advantages over chemotherapy. Liang (Liang et al., 2020) also indirectly compared the efficacy of different combination therapies of atezolizumab, nivolumab, and pembrolizumab in the first-line treatment of NSCLC. The results showed that the advantage of PD-1/PD-L1 inhibitor monotherapy over chemotherapy was not significant. Nevertheless, PD-1/PD-L1 inhibitor combined with chemotherapy showed significant advantages over chemotherapy in terms of PFS. Alfredo et al. (Alfredo et al., 2019) focused on the differences of immunotherapy combined with chemotherapy compared to chemotherapy alone. Using OS and PFS HRs in 8 included RCTs, they found combination therapy improved clinical benefits over chemotherapy alone, which were consistent to our results. Xu et al. (2021) made comparisons of efficacy and safety of single and double immune checkpoint inhibitors (ICIs)-based first-line treatments for advanced wild-type NSCLC, and they found PD-(L)1 combined with chemotherapy had significant survival benefit compared to chemotherapy alone. Though they reached the same conclusion as ours, they did not compare the efficacy of specific drugs in specific populations. Sheng et al. (2021) compared first-line treatments including chemotherapy, anti-angiogenesis, ICIs, and their combinations in treatment of advanced wild-type NSCLC, which was highly similar to Xu et al. (2021) both in terms of research content and research methods. They also found a combination of ICIs with chemotherapy was the best first-line treatment for advanced wild-type NSCLC. However, compared to our comparison of seven ICIs, they only included three and no subgroup analyses were performed for specific populations. Liu et al. (2020) aimed to identify optimal first-line interventions for advanced NSCLC according to PD-L1 expression using a total of 10 RCTs. According to their results, compared with ICIs or chemotherapy alone, the efficacy of immune combination chemotherapy was better, and the efficacy of ICI was superior to chemotherapy alone in treatment of sq-NSCLC. They made the same conclusion as ours, but again, they only considered pembrolizumab and atezolizumab and didn’t make comparisons among specific populations for sq-NSCLC. 12 RCTs were included in Dafni’s study (Dafni et al., 2019), which aimed to aims to compare the efficacy of treatments including at least one ICI with or without chemotherapy. Based on their results, the combination of chemotherapy with either pembrolizumab or atezolizumab showed consistently higher efficacy than chemotherapy-alone or any other ICI-combination or monotherapy. Petrelli et al. (2021) provided evidence that the addition of immune checkpoint inhibitors to chemotherapy may improve both OS and PFS compared with chemotherapy alone using 9 RCTs, similar to Dafni et al. (2019), they only considered two PD(L)1 drugs, pembrolizumab and atezolizumab, and did not do subgroup analyses for specific drugs.

### Novelty of our study

Summarizing the above published NMAs, we can draw the following conclusions:

First and most importantly, in previous NMAs targeted on advanced NSCLC, the researchers took HR as a measure of efficacy, and used the gemtc package under the Bayesian framework or the netmeta package under the frequentist framework. This method is simple and requires minimal effort. However, it is worth noting that the HR is the ratio of efficacy within a specific time frame between treatments calculated using a semi-parametric Cox-PH model. As can be seen from Supplemntary Figure S2, the PH assumption was not valid in this study, that is, the relative efficacy between treatments changed over time. Study duration varied across RCTs (e.g., 3-years data was available from Keynote 407, while only 1-year data was available from Rationale 307), which further limited the use of the Cox-PH model. For example, OS HR for pembrolizumab in combination with chemotherapy *versus* chemotherapy is 0.64 at 18th month (Paz-Ares et al., 2018), while equals to 0.59 at 36th month in Keynote 407 (Paz-Ares et al., 2020). As Wang L (Wang L et al., 2021) did, it is not enough to adopt the PH model, the results need to be verified by the non-PH model. That is, results in existing NMA studies using constant HR models targeting on wild-type advanced NSCLC may be not reliable. In our study, we considered a variety of models, including both the traditional PH model and FP models with non-constant HRs. In addition, as mentioned by Huang and Kun.2018, non-parametric RMST model was an alternative robust and clinically interpretable summary measure for efficacy, considering that the parametric model had a certain bias. Thus, we used the RMST model to verify the results. Furthermore, we used survival time as a measure of efficacy, which can more clearly see the survival benefits brought by different drugs to patients. At the same time, we extrapolated survival curves to predict the long-term efficacy which was lack in previous studies.

Secondly, these studies were basically focused on one question, that is, the efficacy of immunotherapy combined with chemotherapy compared with chemotherapy alone, few articles discussed the relative efficacy between immunotherapies for advanced wild-type sq-NSCLC. Compared to them, we systematically compared these PD-(L)1 inhibitors, and 4 PD-(L)1 inhibitors were firstly considered by us, including sugemalimab, camrelizumab, tislelizumab, toripalimab.

Thirdly, previous studies have not analyzed the effects of specific drugs on populations in specific regions. The differences in the baseline characteristics of the population will greatly affect the accuracy of the results. Therefore, our study focused on a specific population and compared the effects of different immune target inhibitors on the Chinese population for the first time.

To conclude, this study was the first to indirectly compare PD-(L)1 inhibitors that had been on the market or about to enter the market in China for the first line treatment of advanced sq-NSCLC, and 4 PD-(L)1 inhibitors were firstly compared by us. We compared the efficacy of these treatments and extrapolate them to long-term survival benefits. We validated the robustness of results against different assumptions of HRs (time invariant vs. time varying), together with non-parametric RMST model, which had not been addressed by previous NMAs. This analysis was necessary given that non-PH were detected in the most included trial of our study. (Jotte et al., 2020; Paz-Ares et al., 2020; Wang et al., 2021; Wang et al., 2021; Zhou et al., 2022; Zhou et al., 2021). Furthermore, our subgroup analysis of PD-(L)1 expression, smoking, gender, ECOG performance status, age and disease stage can provide reference for clinical precision medicine. Finally, this study summarized the evidence for and safety, which could provide some support for decision-making for drug use.

### Limitations of our study

Our study also had some limitations. First, the bias of different baseline information among clinical trials could not be ignored. Different baseline characteristics including age, gender and clinical stage may lead to data lacking comparability. Second, indirect comparison enlarged the variance, which might result in non-significant therapeutic effects and even remove the differences between studies. As a result, the conclusions on the ranking of therapeutic effects were relatively conservative. Third, the OS data of some clinical trials were immature and we did not distinguish between types of SAEs, which may cause some bias. Thus, improved trial data were needed to make the results more realistic. Finally, we chose PFS data only from Blinded Independ Review Committee (BIRC) for unblinded trials, such as Orient-12. PFS data for other regimens were from BIRC, and when data of blinded trials was not available, results from investigator review were used after considering that in randomized double-blind double-dummy trials, investigator assessment was indistinguishable from BIRC assessment (Dodd et al., 2008).

## Conclusion

Based on the comprehensive results of this study, sugemalimab is recommended for the first-line treatment of advanced sq-NSCLC in China in terms of PFS and OS benefit. Although the conclusions of this study are conservative, these findings provide relevant evidence for clinical decision-making and health insurance. Future clinical trials with more comparable baseline information or even direct head-to-head comparison are anticipated, which can fill the lack of evidence on the efficacy of PD-(L)1 in the treatment of sq-NSCLC in China.

## Data Availability

The original contributions presented in the study are included in the article/Supplementary Material, further inquiries can be directed to the corresponding author.
