# Supplementary material for: Identifying optimal PD-1/PD-L1 inhibitors in first-line treatment of patients with advanced squamous non-small cell lung cancer in China: Updated systematic review and network meta-analysis
Source: Front Pharmacol. 2022 Sep 29;13:910656. doi: 10.3389/fphar.2022.910656 (PMC9558711; doi:10.3389/fphar.2022.910656)
Supplement: Supplementary file 2 [file DataSheet1.docx]

Supplementary appendix 1

[Method S1 Search Strategies 2](#_Toc32547)

[Table S1 Basic Characteristics of Included Studies 3](#_Toc913)

[Figure S1 Summary of Results from Assessment of Studies Using the Cochrane Risk of Bias Tool 5](#_Toc28297)

[Figure S2 Log Cumulative Hazards Plots for Overall Survival, Progression Free Survival 6](#_Toc14988)

[Figure S3 Fitted Curves of Overall Survival and Progression Free Survival of Standard Chemotherapy Regimens by Minimum-AIC Fractional Polynomial Models, Royston-Parmar, Restricted Cubic Spline and All Standard Distribution Models 8](#_Toc3761)

[Table S2 AICs for Royston-Parmar and Standard Parametric Models of Overall Survival, Progression Free Survival for Anchor Treatment 8](#_Toc3300)

[Figure S4 Fitted Survival Curves of All FP Models for Overall Survival, Progression Free Survival 9](#_Toc30930)

[Figure S5 Final Fitted Survival Curves of Overall Survival, Progression Free Survival 11](#_Toc15907)

[Table S3 AICs for all first-order Fractional Polynomial Models of Overall Survival, Progression Free Survival in network meta-analysis 11](#_Toc23763)

[Table S4 HR Related Parameter for Fractional Polynomial Models 12](#_Toc22282)

[Figure S6 Treatment Ranking Probabilities for Overall Survival, Progression Free Survival, and Severe Adverse Events. 13](#_Toc16127)

[Figure S7 Subgroup analysis results for Overall Survival, Progression Free Survival. 16](#_Toc14084)

# **Method S1** **Search Strategies**

PubMed

| No | Items |
| --- | --- |
| #1 | PD-1[Title/Abstract] OR PD-L1[Title/Abstract] OR Immunotherapy[Title/Abstract] |
| #2 | sq[Title/Abstract] OR Squamous[Title/Abstract] |
| #3 | NSCLC[Title/Abstract] OR Non-small cell lung cancer[Title/Abstract] |
| #4 | #1 AND #2 AND #3 |
| #5 | Animals[Title] OR Human[Title] |
| #6 | #4 NOT #5 |
| #7 | Clinical Trial[Publication Type] OR Randomized Controlled Trial[Publication Type] |
| #8 | #6 AND #7 |

Embase

| #1 | 'Squamous Non-small cell lung cancer'/exp OR ('Non-small cell lung cancer') NEAR/3 (Squamous*OR cancer OR cancers* OR cancerous* OR tumor* OR tumour*)):ab,ti,kw,tn |
| --- | --- |
|  |  |
|  |  |
| #2 | Atezolizumab'/exp OR (Atezolizumab OR "IMpower 131" ):ab,ti,kw,tn |
| #3 | Pembrolizumb'/exp OR (Pembrolizumb OR "Keynote407" ):ab,ti,kw,tn |
| #4 | Sintilimab'/exp OR (Sintilimab OR "Orient 12" ):ab,ti,kw,tn |
| #5 | Camrelizumb'/exp OR (Camrelizumb OR "SHR-1210-Ⅲ-307" ):ab,ti,kw,tn |
| #6 | Tislelizumab'/exp OR (Tislelizumab OR "Rationale307" ):ab,ti,kw,tn |
| #7 | Sugemalimab'/exp OR (Sugemalimab OR "Gemstone302" ):ab,ti,kw,tn |
| #8 | Toripalimab'/exp OR (Toripalimab OR "CHOICE-01" ):ab,ti,kw,tn |
| #9 | #2 OR #3 OR #4 OR #5 OR #6 #7 #8 |
| #10 | #1 AND #9 |
| #11 | crossover procedure':de OR 'double-blind procedure':de OR 'randomized controlled trial':de OR 'single-blind procedure':de OR 'drug therapy'/lnk OR (random* OR factorial* OR crossover* OR cross NEXT/1 over* OR placebo* OR doubl* NEAR/1 blind* OR singl* NEAR/1 blind* OR assign* OR allocat* OR volunteer*):de,ab,ti |
|  |  |
|  |  |
|  |  |
|  |  |
| #12 | #10 AND #11 |
| #13 | ('animal'/exp OR 'animal experiment'/exp) NOT 'human'/exp |
| #14 | #12 NOT #13 |

ClinicalTrials

https://clinicaltrials.gov/ct2/results/refine?show_xprt=Y

(Squamous Non-small-cell Lung Cancer) AND (Pembrolizumb OR Atezolizumab OR Sintilimab OR Camrelizumb OR Tislelizumab OR Sugemalimab OR Toripalimab OR Immunotherapy) AND (Phase 3)

# **Table S1 Basic Characteristics of Included Studies**

| study | NCT | treatment | | stage | Sample size | | age | | sex(male/female) | | region(/race) | | ECOG score(0,1,≥2，missing) | | Smoking status(Never smoked/Current or former smoker/missing) | | PD-L1 status(＜1%/≥1%/1-49%/≥50%（could not be evaluated/unknown）) | |
| --- | --- | --- | --- | --- | --- | --- | --- | --- | --- | --- | --- | --- | --- | --- | --- | --- | --- | --- |
|  |  | Intervention arm（arm 1） | Control arm（arm 2） |  | arm 1 | arm 2 | arm 1 | arm 2 | arm 1 | arm 2 | arm 1 | arm 2 | arm 1 | arm 2 | arm 1 | arm 2 | arm 1 | arm 2 |
| keynote-407 | NCT02775435 | Pembrolizumab+ control arm | normal saline or nab-paclitaxel +carboplatin | Ⅳ | 278 | 281 | 65 | 65 | 220/58 | 235/46 | global | global | 73/205 | 90/191 | 22/256 | 19/262 | 95/176/103/73 | 99/177/104/73 |
| Orient-12 | NCT03629925 | Sintilimab + control arm | gemcitabine and either cisplatin or carboplatin+placedo | ⅢB，Ⅳ | 179 | 178 | 64 | 62 | 163/16 | 164/14 | Chinese | Chinese | 30/149 | 22/156 | 24/155 | 31/147 | 59/120/62/58 | 63/115/52/63 |
| CameL-sq | NCT03668496 | Camrelizumb + control arm | carboplatin and paclitaxel | Ⅳ | 193 | 196 | / | / | / | / | Chinese | Chinese | / | / | / | / | / | / |
| Rationale-307 | NCT03594747 | Tislelizumab + control arm | carboplatin and paclitaxel | ⅢB，Ⅳ | 120 | 121 | 60 | 62 | 107/13 | 111/10 | Chinese | Chinese | 31/89 | 32/89 | 24/96 | 23/98 | 48/-/30/42 | 49/-/31/41 |
| Gemstone-302 | NCT03789604 | Sugemalimab + control arm | pemetrexed, paclitaxel and carboplatin | Ⅳ | 320 | 159 | 62 | 64 | 254/66 | 129/30 | Chinese | Chinese | 59/261 | 25/134 | 88/232 | 40/119 | 124/196/-/- | 64/95/-/- |
| CHOICE-01 | NCT03856411 | Toripalimab(200 mg/3 weeks) + control arm | placebo,nab-paclitaxel and carboplatin | Ⅳ | 309 | 156 | 63 | 61 | 247/62 | 130/26 | Chinese | Chinese | 66/243 | 36/120 | 96/213 | 49/107 | 108/201/-/- | 53/103/-/- |
| Impower-131 | NCT02367794 | Atezolizumab + control arm | nab-paclitaxel and carboplatin | Ⅳ | 343 | 340 | 65 | 65 | 280/63 | 277/163 | global | global | 115/227/0/1 | 110/229/0/1 | 32/311/0 | 23/316/1 | 160/182/-/47 | 171/169/-/44 |

# **Figure S1 Summary of Results from Assessment of Studies Using the Cochrane Risk of Bias Tool**


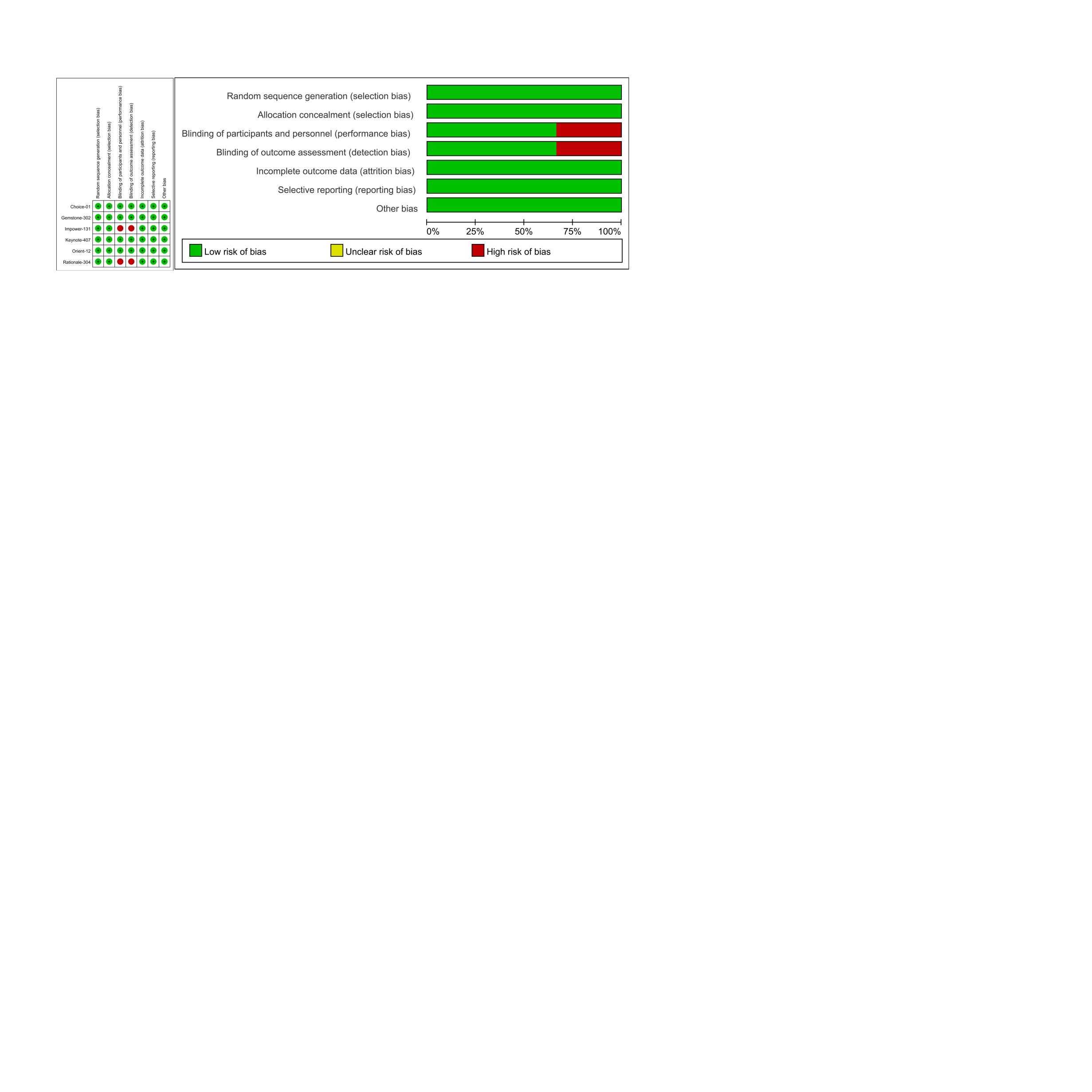


# **Figure S2 Log Cumulative Hazards Plots for Overall Survival, Progression Free Survival**

Overall survival


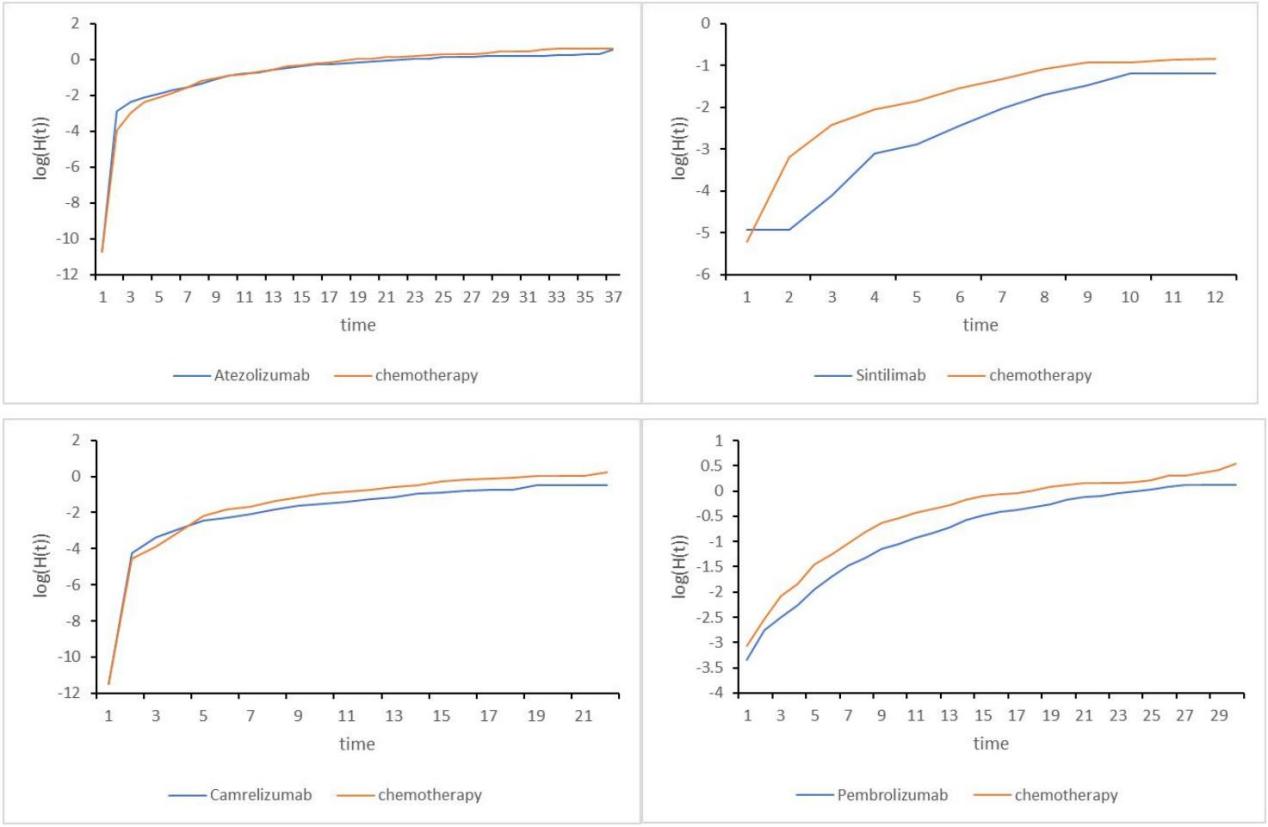


*: Sugemalimab, toripalima and tislelizumab is lack of OS curve for sq-NSCLC

Progression-free survivaL


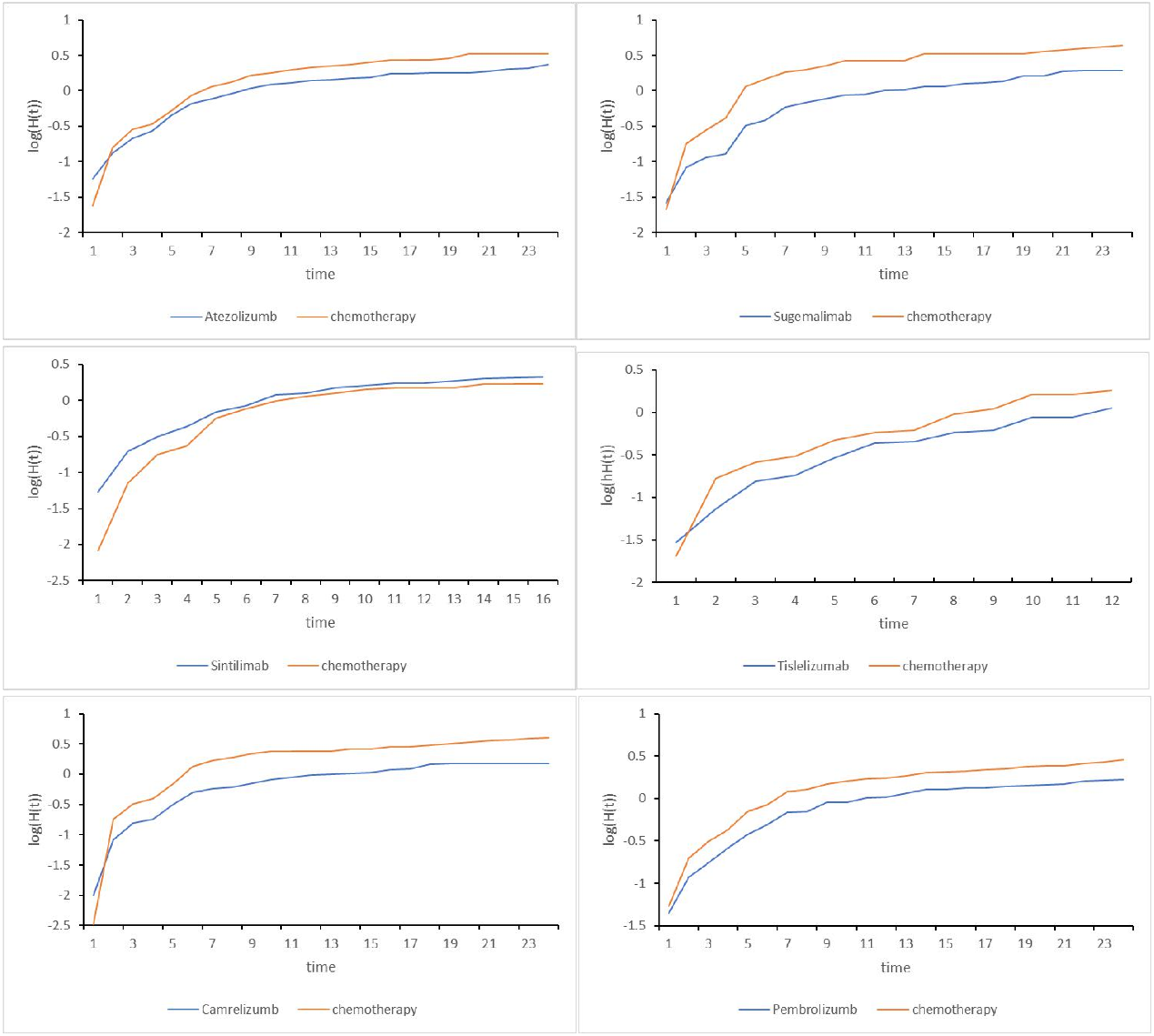


*: Toripalimab is lack of PFS curve for sq-NSCLC

# **Figure S3** **Fitted Curves of Overall Survival and Progression Free Survival of Standard Chemotherapy Regimens by Minimum-AIC Fractional Polynomial Models, Royston-Parmar, Restricted Cubic Spline and All Standard Distribution Models**

| 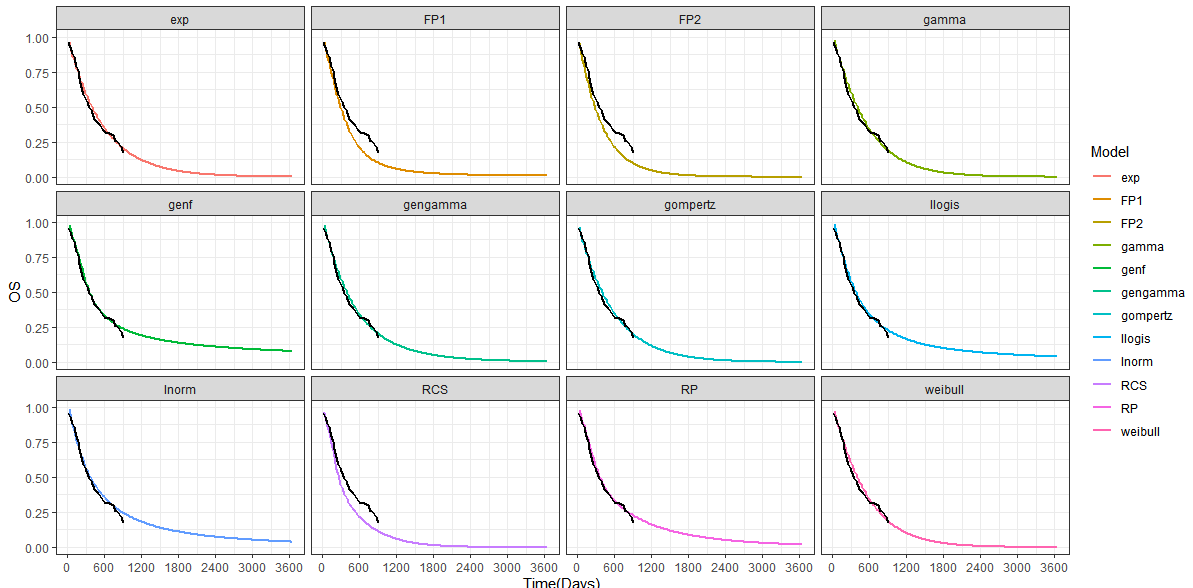 |
| --- |
| 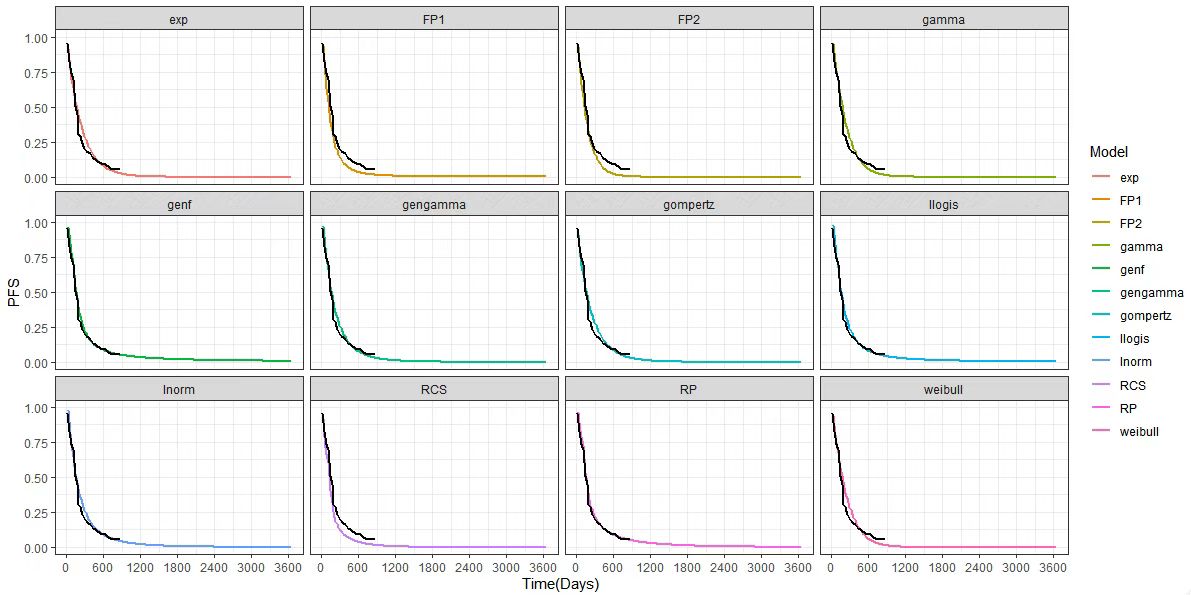 |

# **Table S2** **AICs for Royston-Parmar and Standard Parametric Models of Overall Survival, Progression Free Survival for Anchor Treatment**

| OS | | | | | PFS | | | | |
| --- | --- | --- | --- | --- | --- | --- | --- | --- | --- |
| Royston-Parmar | | | Standard Parametric Models | | Royston-Parmar | | | Standard Parametric Models | |
| K | Scale | AIC | model | AIC | K | Scale | AIC | model | AIC |
| 0 | hazard | 567.52 | exponential | 567.31 | 0 | hazard | 279.19 | exponential | 283.32 |
|  | odds | 564.26 | weibull | 567.52 |  | odds | 248.43 | weibull | 279.19 |
|  | normal | 575.27 | gamma | 566.84 |  | normal | 260.30 | gamma | 273.03 |
| 1 | hazard | 567.88 | log-normal | 575.27 | 1 | hazard | 257.10 | log-normal | 260.3 |
|  | odds | 564.68 | gompertz | 569.28 |  | odds | 249.75 | gompertz | 284.8 |
|  | normal | 565.45 | log-logitics | 564.26 |  | normal | 257.52 | log-logitics | 248.43 |
| 2 | hazard | 562.92^*^ | gengamma | 567.47 | 2 | hazard | 242.70 | gengamma | 259.25 |
|  | odds | 563.92 | genF | 564 |  | odds | 245.28 | genF | 241.53 |
|  | normal | 564.09 |  |  |  | normal | 244.52 |  |  |
| 3 | hazard | 562.92 |  |  | 3 | hazard | 244.17 |  |  |
|  | odds | 563.92 |  |  |  | odds | 242.13 |  |  |
|  | normal | 564.09 |  |  |  | normal | 241.44 |  |  |
| 4 | hazard | 565.34 |  |  | 4 | hazard | 239.13 |  |  |
|  | odds | 566.32 |  |  |  | odds | 239.12 |  |  |
|  | normal | 566.31 |  |  |  | normal | 239.48 |  |  |
| 5 | hazard | 565.49 |  |  | 5 | hazard | 237.83^*^ |  |  |
|  | odds | 565.84 |  |  |  | odds | 239.62 |  |  |
|  | normal | 565.02 |  |  |  | normal | 241.82 |  |  |

*: the smallest AIC among all models

# **Figure S4 Fitted Survival Curves of All FP Models for Overall Survival, Progression Free Survival**

Overall survival


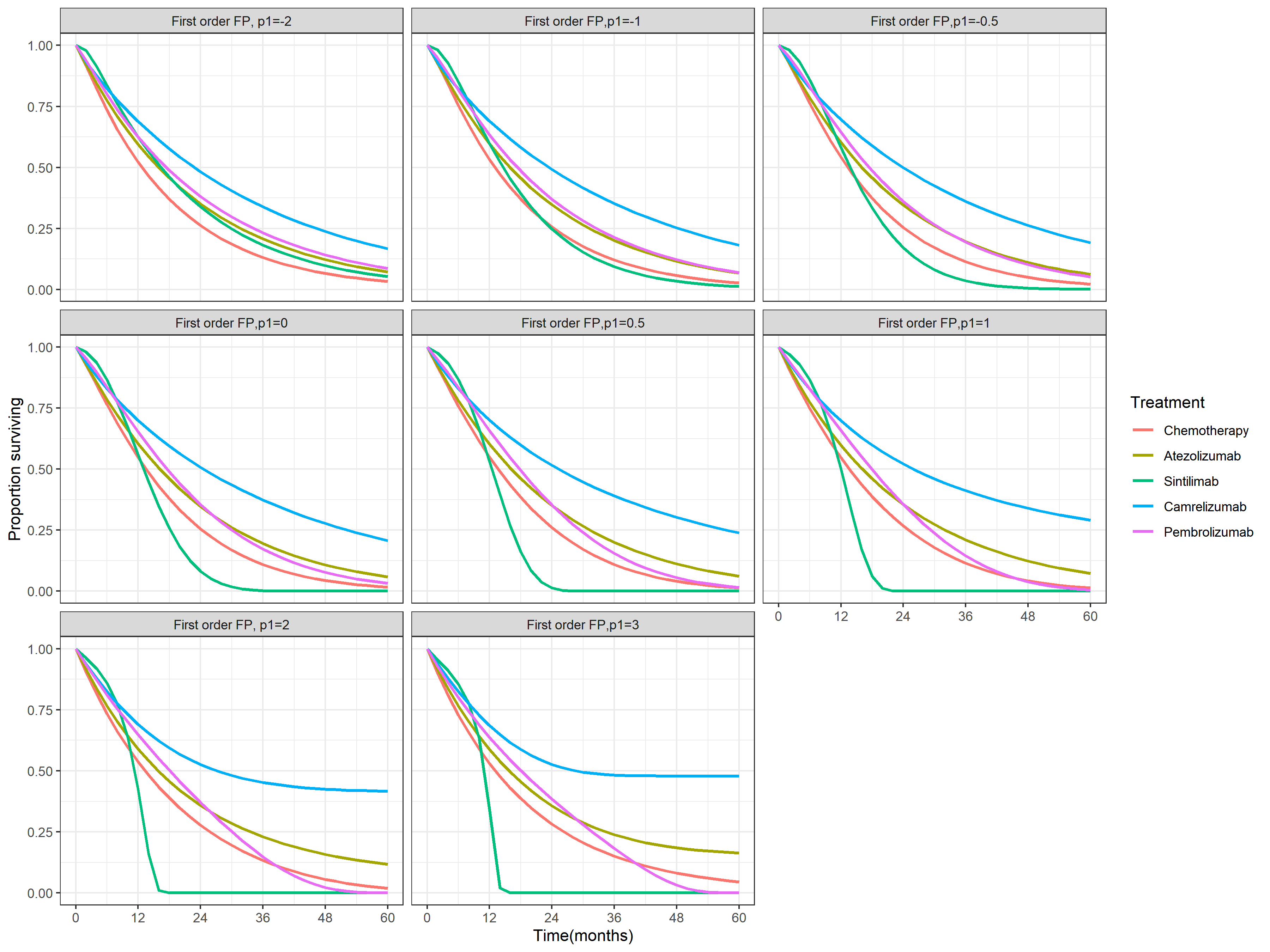


Progression-free survival


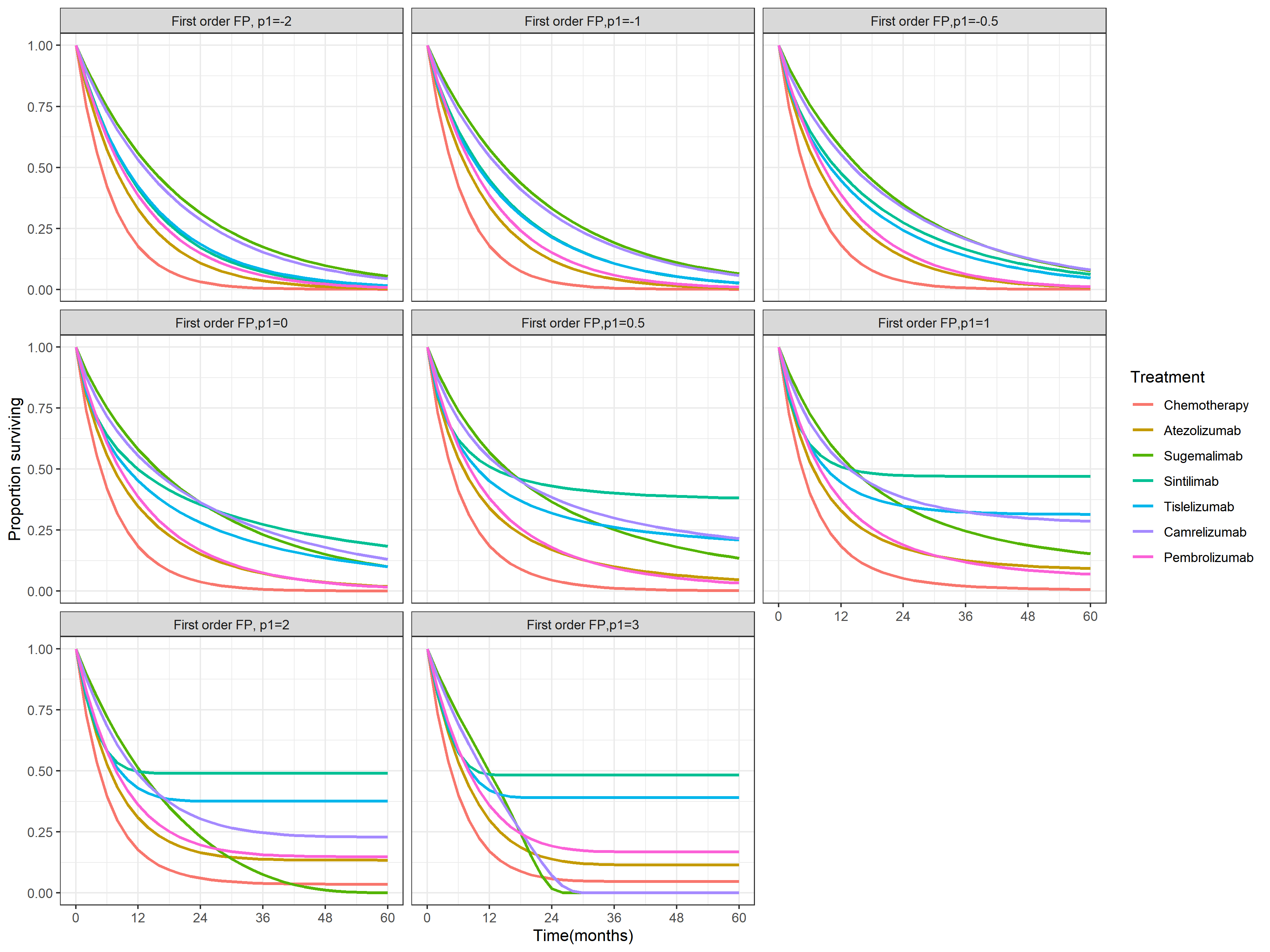


Fitted Survival Curves of Overall survival (Considered sintilimab) for best fitted model


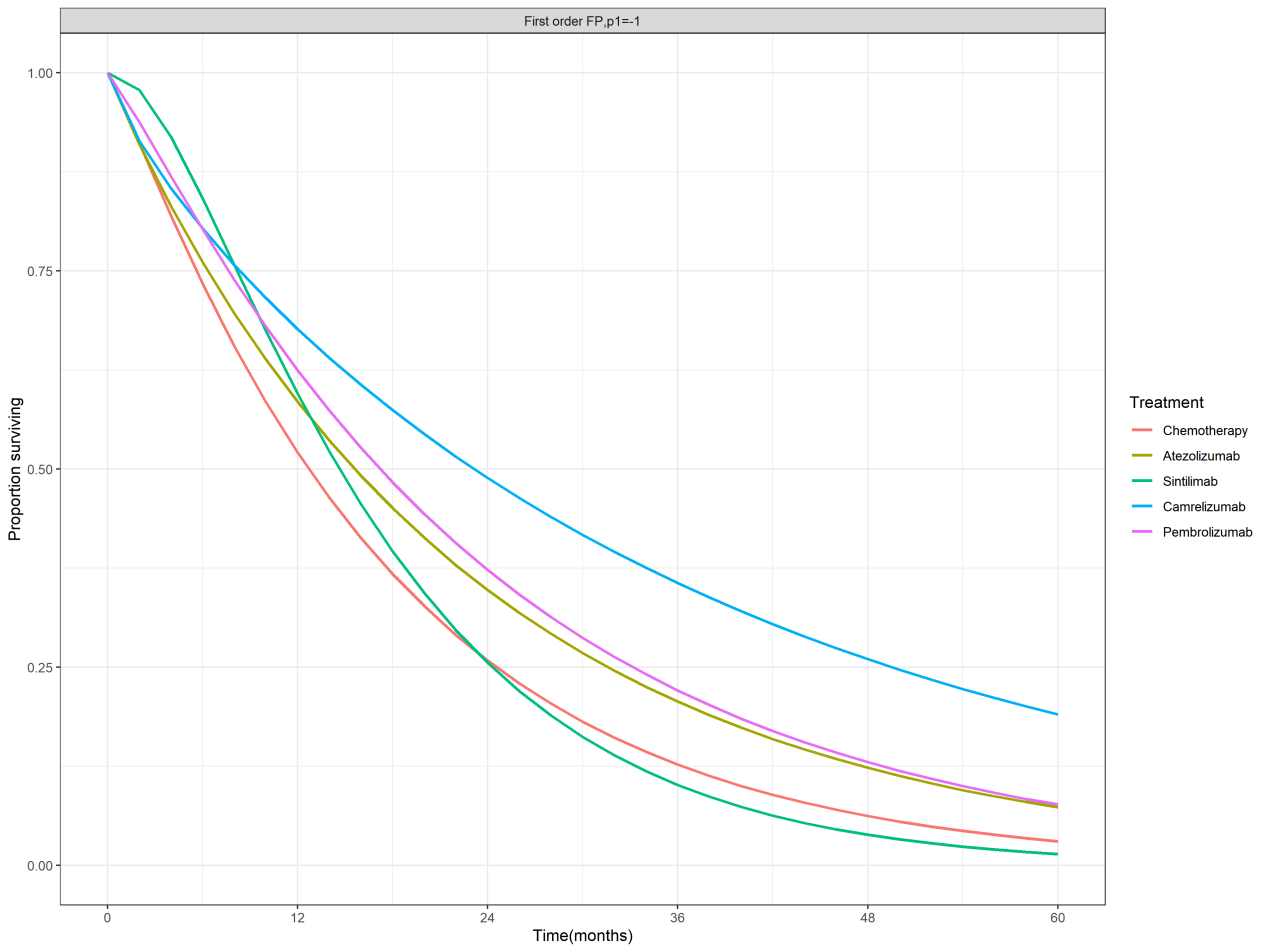


# **Figure S5 Final Fitted Survival Curves of Overall Survival, Progression Free Survival**

| 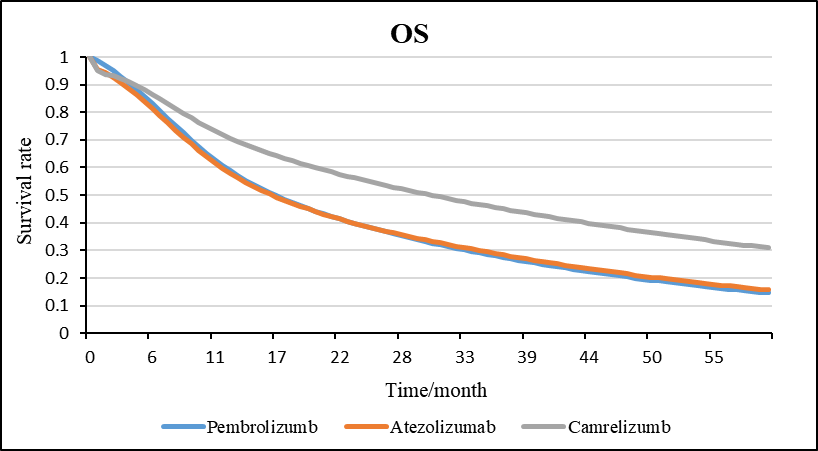 |
| --- |
| 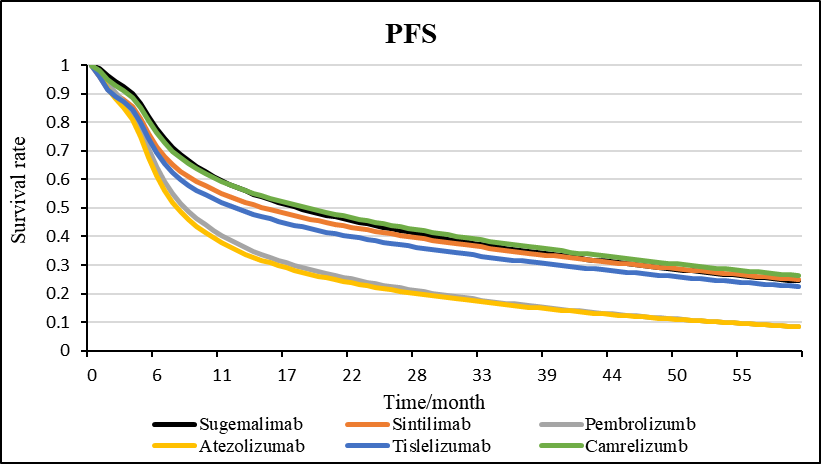 |

# **Table S3** **AICs for all first-order Fractional Polynomial Models of Overall Survival, Progression Free Survival in network meta-analysis**

| OS | | PFS | |
| --- | --- | --- | --- |
| power | AIC | power | AIC |
| -2 | 742.01 | -2 | 1472.51 |
| -1^*^ | 734.29 | -1 | 1455.32 |
| -0.5 | 735.05 | -0.5^*^ | 1453.20 |
| 0 | 741.20 | 0 | 1459.16 |
| 0.5 | 749.77 | 0.5 | 1469.44 |
| 1 | 756.97 | 1 | 1478.22 |
| 2 | 764.77 | 2 | 1486.44 |
| 3 | 768.28 | 3 | 1490.68 |

*: the smallest AIC among all first-order models

# **Table S4** **HR Related Parameter for Fractional Polynomial Models**

| Parameter | treatment | mean | 95%CI | |
| --- | --- | --- | --- | --- |
| PFS_d_0_ VS chemotherapy | Sugemalimab | -1.222 | -2.117 | -0.315 |
|  | Sintilimab | -1.429 | -2.127 | -0.784 |
|  | Pembrolizumb | -0.651 | -1.075 | -0.225 |
|  | Atezolizumab | -0.683 | -1.116 | -0.263 |
|  | Camrelizumb | -1.373 | -1.987 | -0.749 |
|  | Tislelizumab | -1.315 | -2.312 | -0.333 |
| PFS_d_1_ VS chemotherapy | Sugemalimab | 0.172 | -1.423 | 1.775 |
|  | Sintilimab | 1.333 | 0.109 | 2.549 |
|  | Pembrolizumb | 0.144 | -0.645 | 0.909 |
|  | Atezolizumab | 0.477 | -0.285 | 1.254 |
|  | Camrelizumb | 0.707 | -0.365 | 1.749 |
|  | Tislelizumab | 1.268 | -0.363 | 2.9 |
| OS _d_0_ VS chemotherapy | Pembrolizumb | -0.296 | -0.58 | -0.011 |
|  | Atezolizumab | -0.334 | -0.583 | -0.087 |
|  | Camrelizumb | -0.863 | -1.334 | -0.399 |
| OS_d_1_ VS chemotherapy | Pembrolizumb | -0.165 | -1.213 | 0.845 |
|  | Atezolizumab | 0.7 | -0.227 | 1.639 |
|  | Camrelizumb | 1.649 | -0.274 | 3.63 |

# **Figure S6 Treatment Ranking Probabilities for Overall Survival, Progression Free Survival, and Severe Adverse Events.**

Overall survival (Cox Proportional Hazards Model)

Progression free survival (Cox Proportional Hazards Model)

Severe Adverse Events

Progression free survival rank (Restricted Mean Survival Time model)


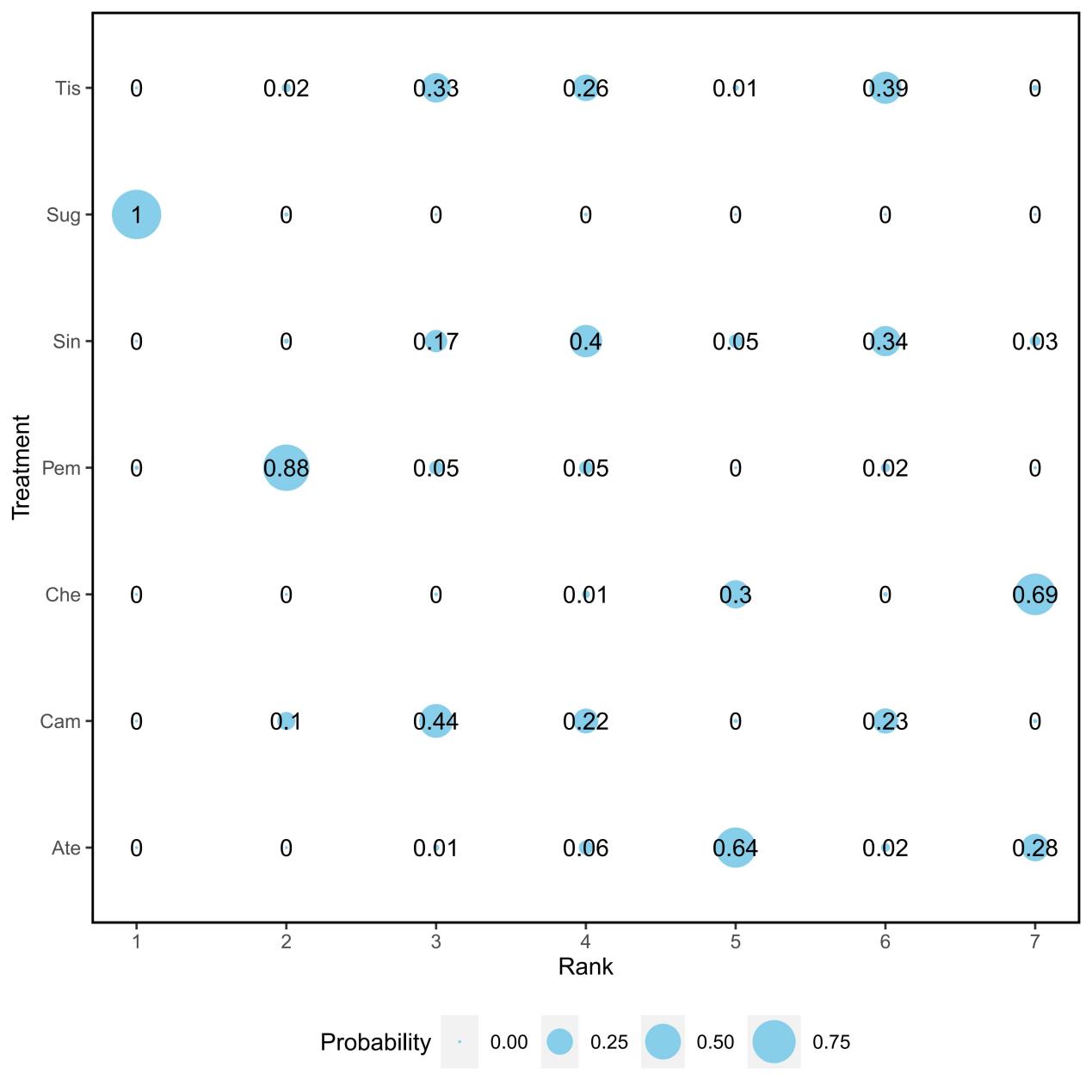


Overall survival rank (Restricted Mean Survival Time model)


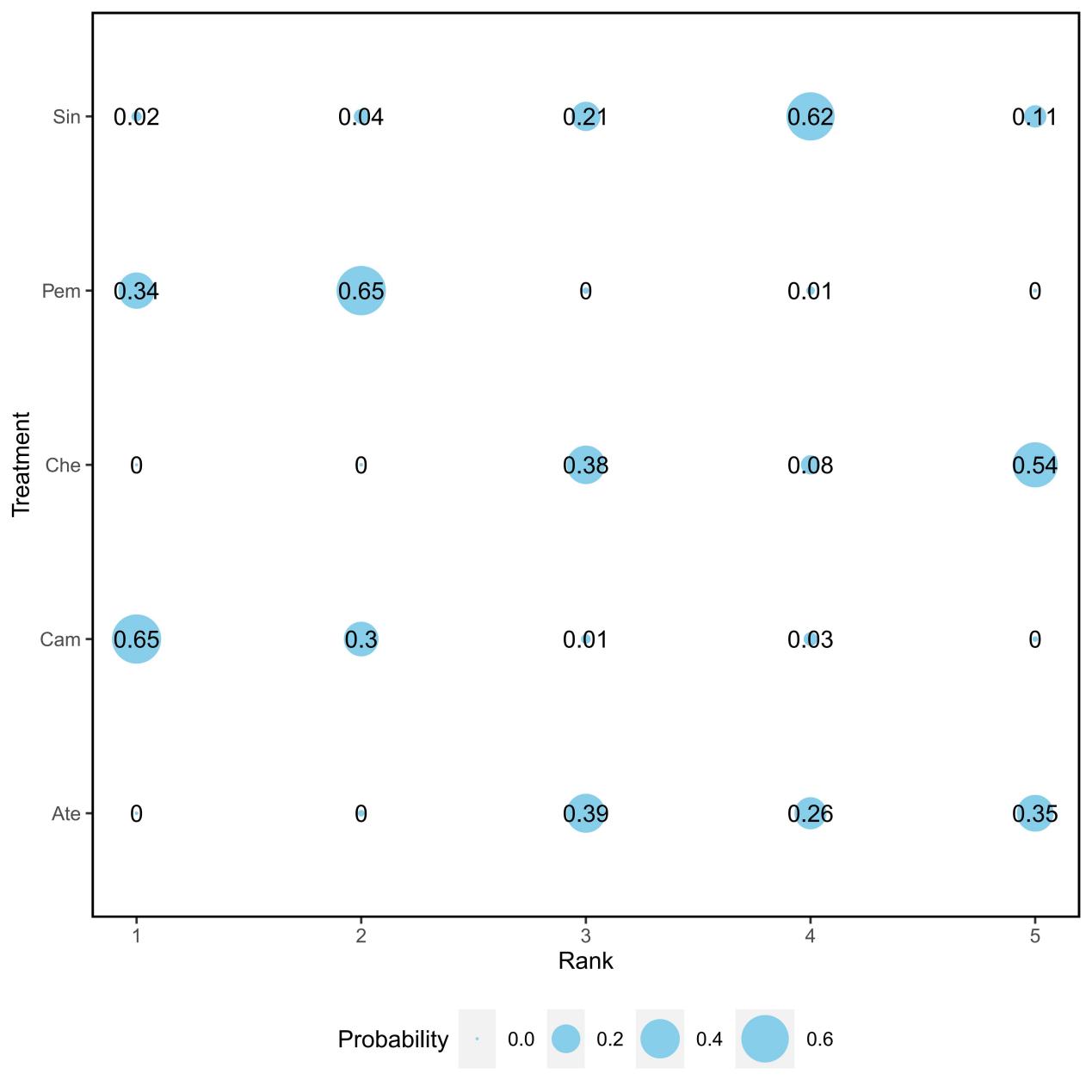


# **Figure S7 Subgroup analysis results for Overall Survival, Progression Free Survival.**

eFigure 7A Subgroup for Overall survival (Using Cox Proportional Hazards Model)

| PD-L1 <1% | 1% < PD-L1 <50% |
| --- | --- |
| 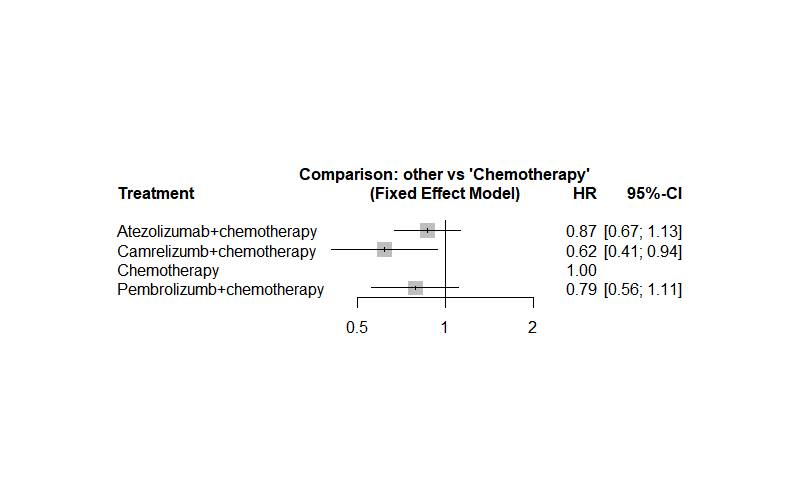 | 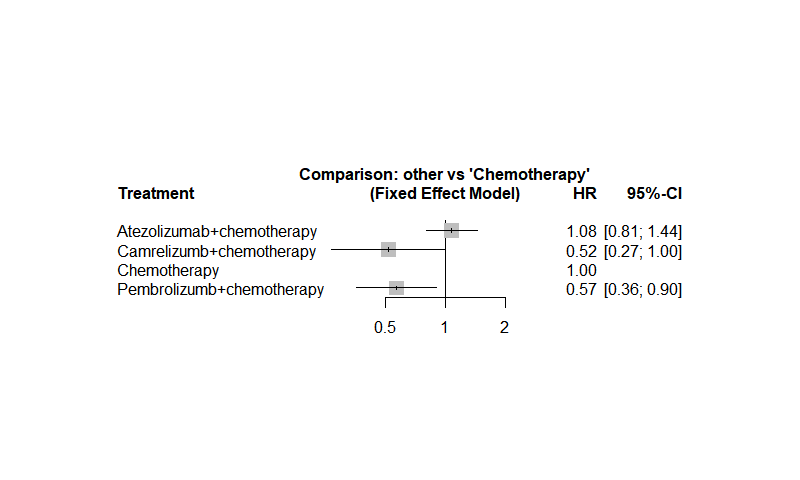 |
| PD-L1 > 50% | Disease stage Ⅳ |
| 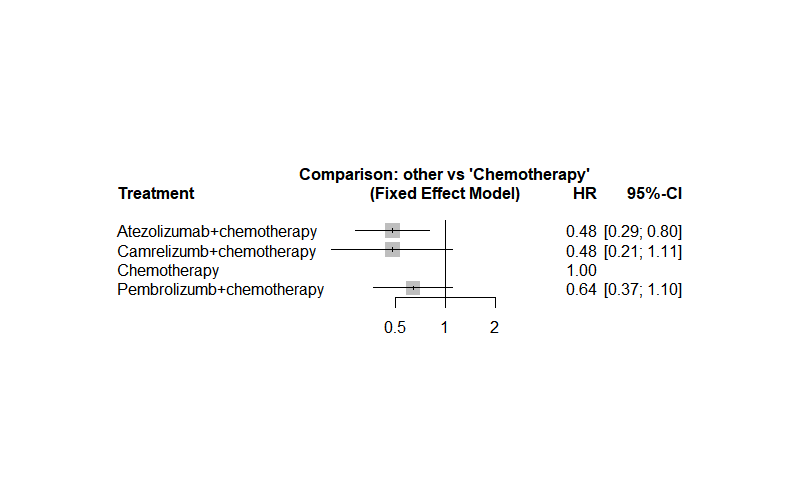 | 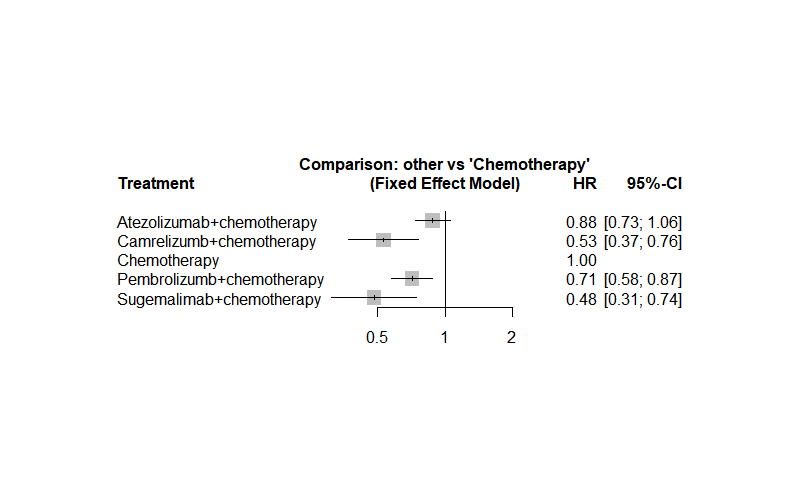 |
| Male | Female |
| 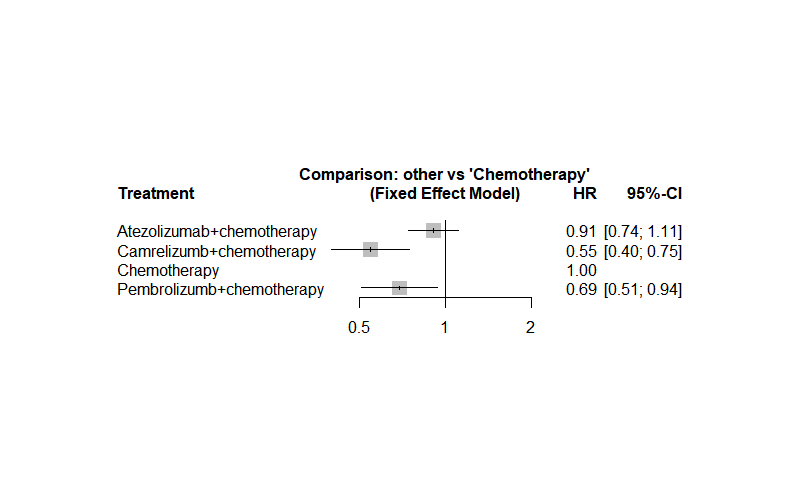 | 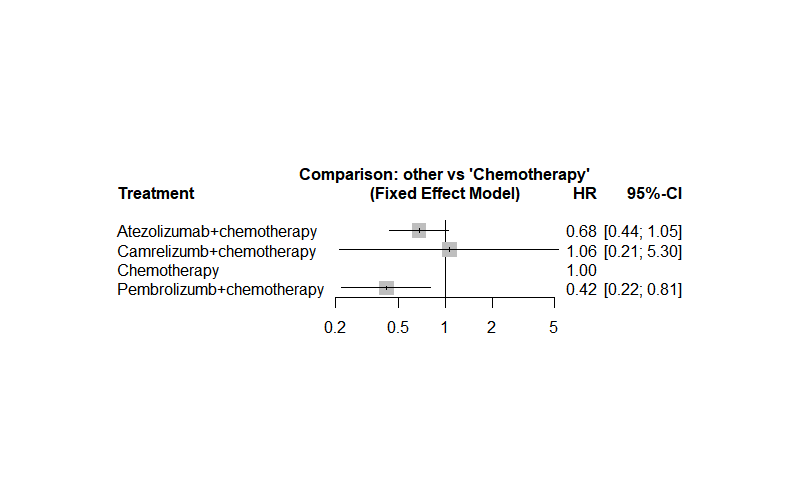 |
| Age < 65 | Age ≥ 65 |
| 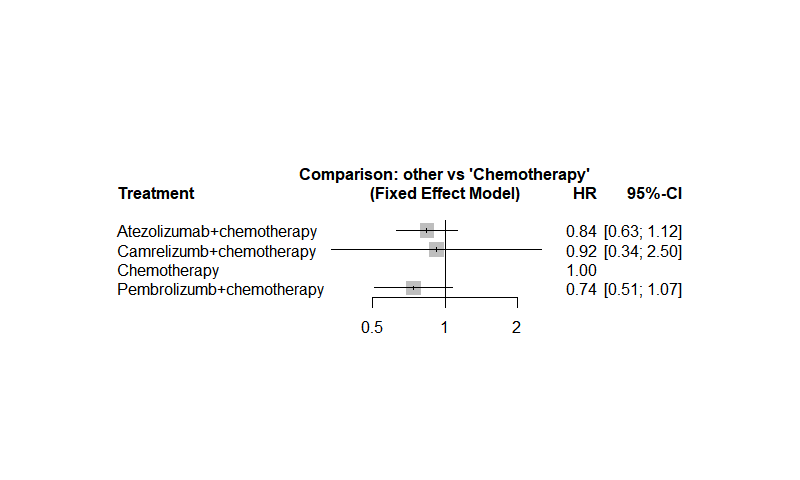 | 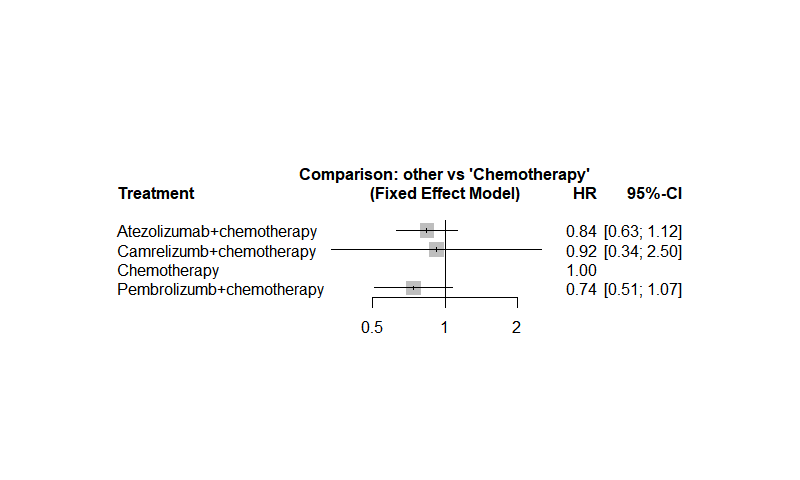 |
| Current or former smoker | Non-smoker |
| 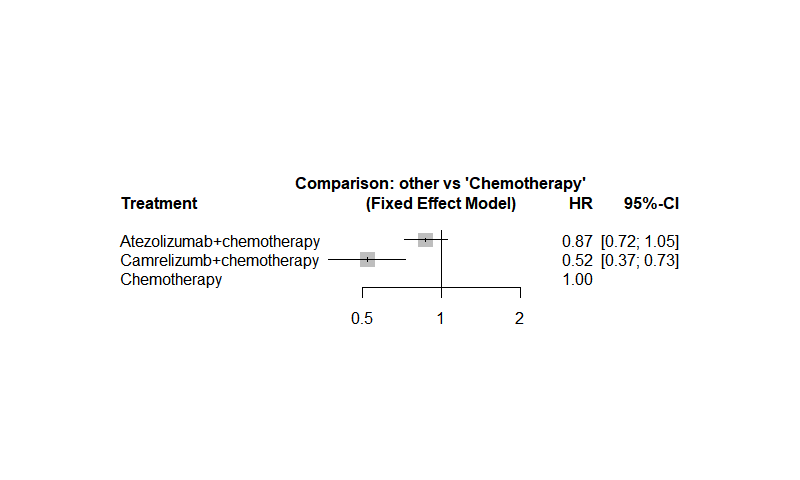 | 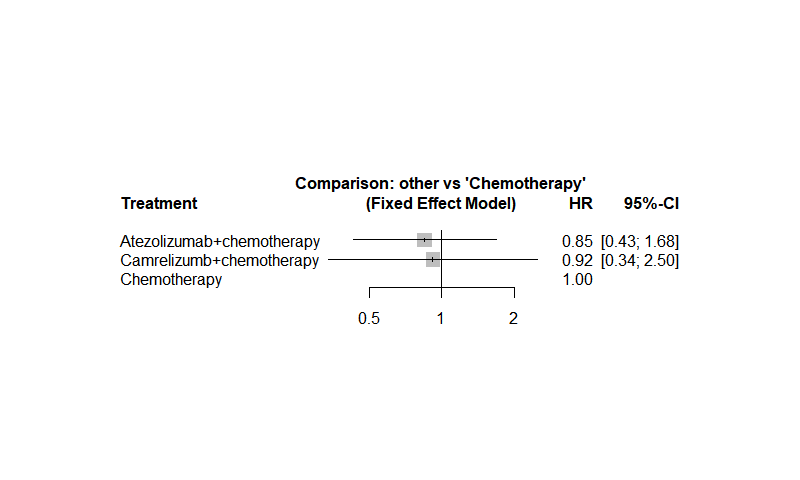 |
| ECOG: 0 | ECOG: 1 |
| 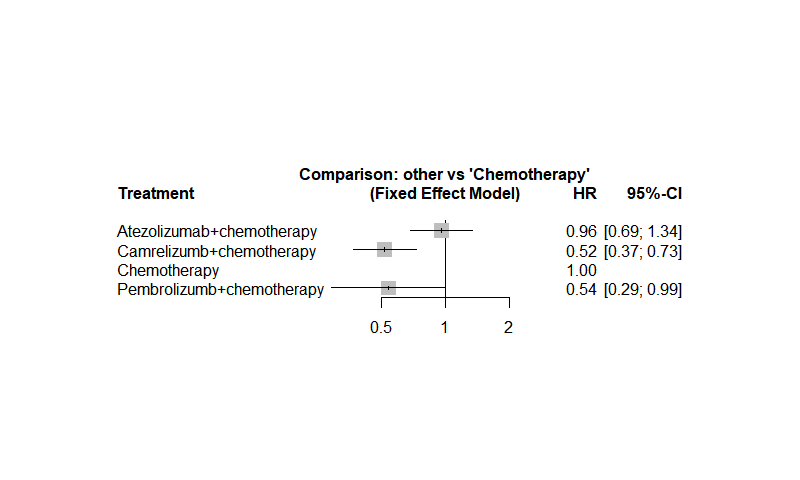 | 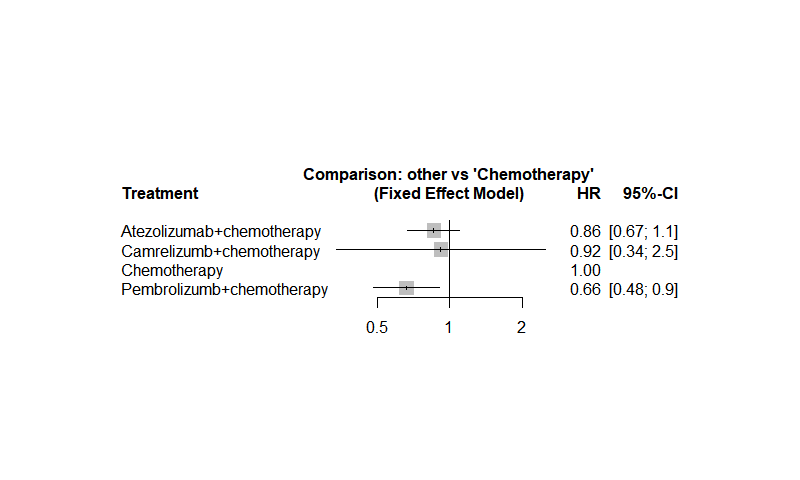 |
| ECOG indicates Eastern Cooperative Oncology Group; HR, hazard ratio; PD-L1, programmed cell death 1 ligand 1. | |

eFigure 7B Subgroup for progression-free survival (Using Cox Proportional Hazards Model)

| PD-L1 <1% | 1% < PD-L1 <50% |
| --- | --- |
| 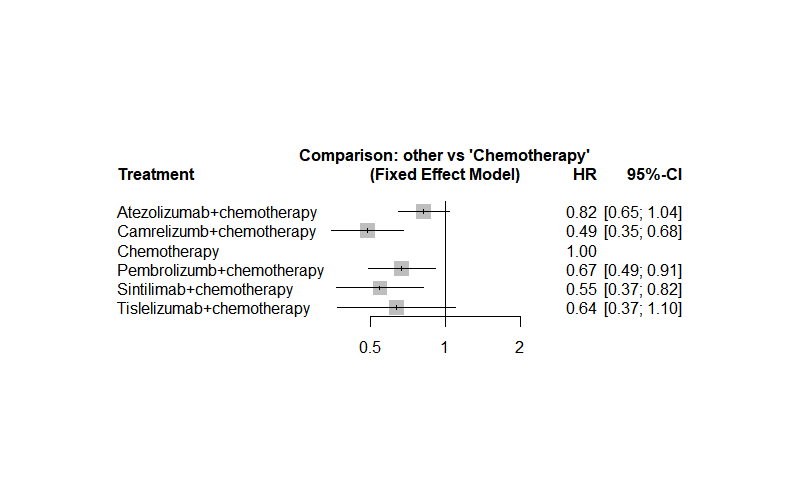 | 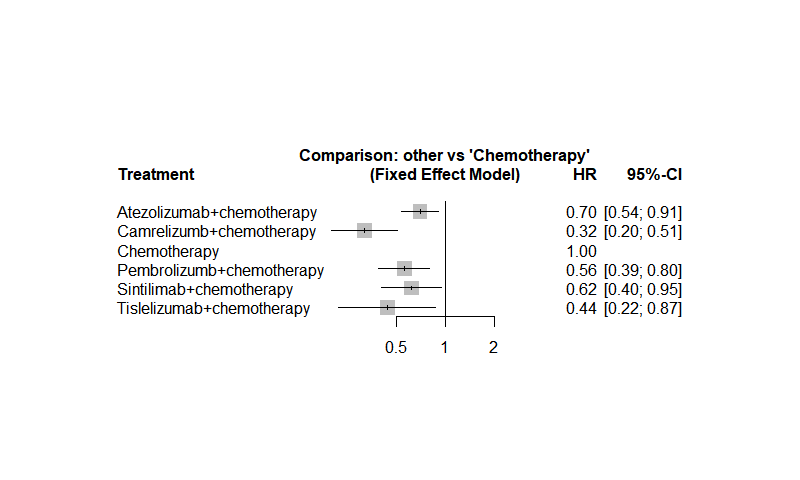 |
| PD-L1 > 50% | Disease stage Ⅳ |
| 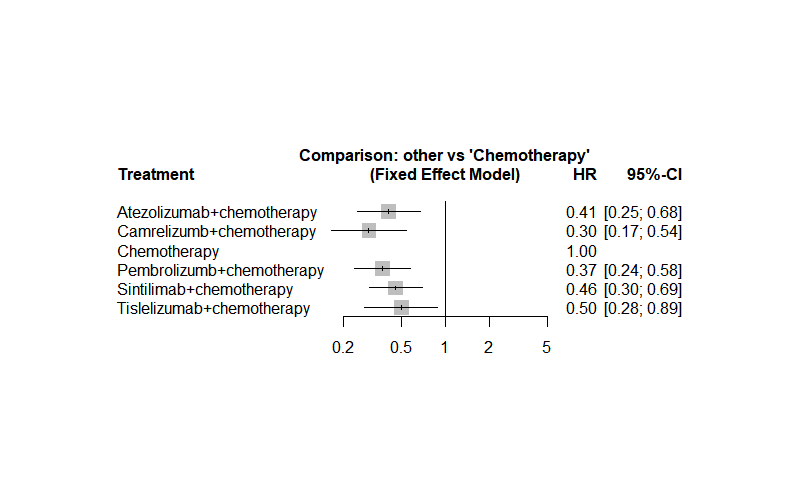 | 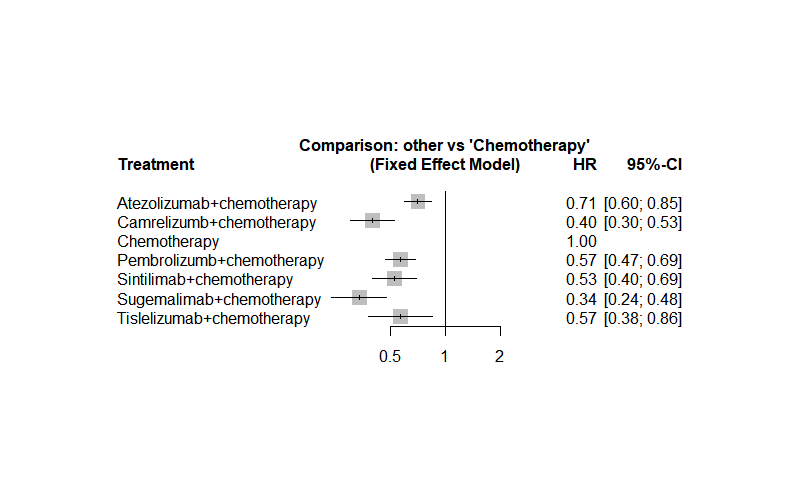 |
| Disease stage III | |
| 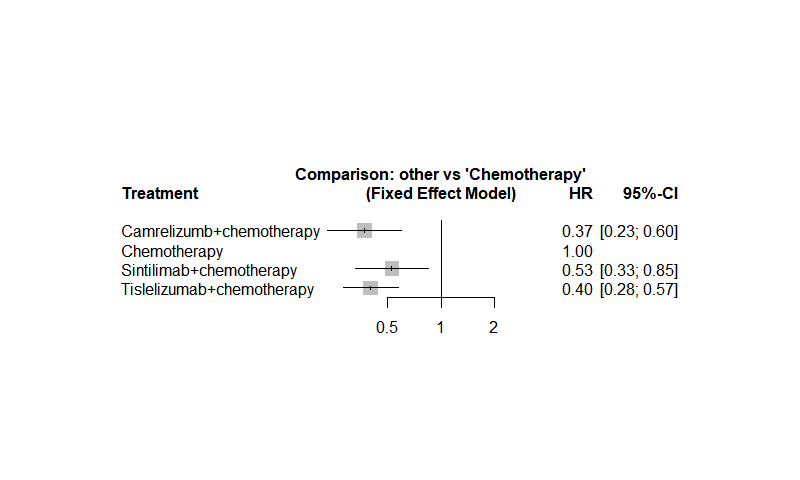 | |
| Male | Female |
| 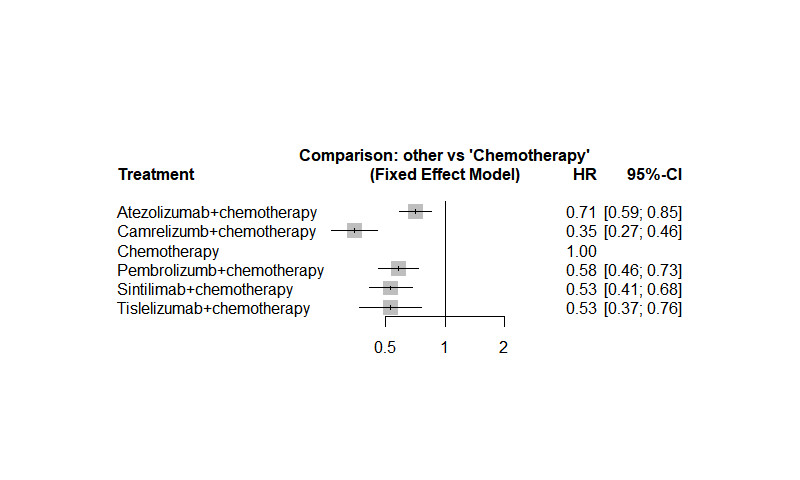 | 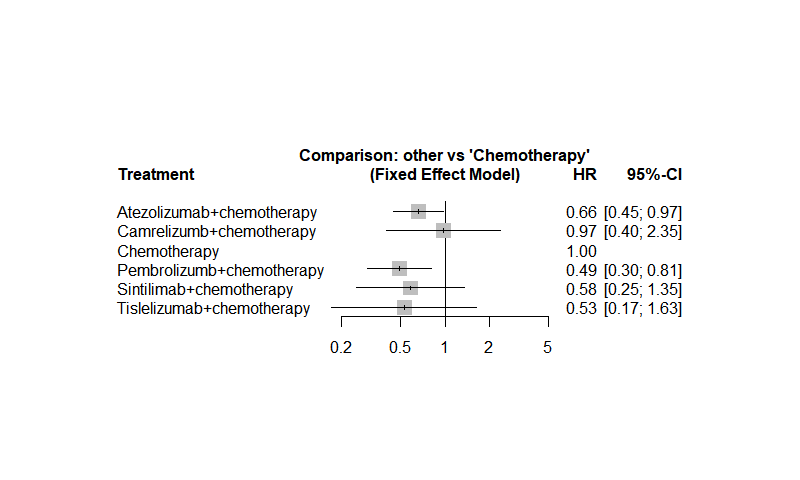 |
| Age < 65 | Age ≥ 65 |
| 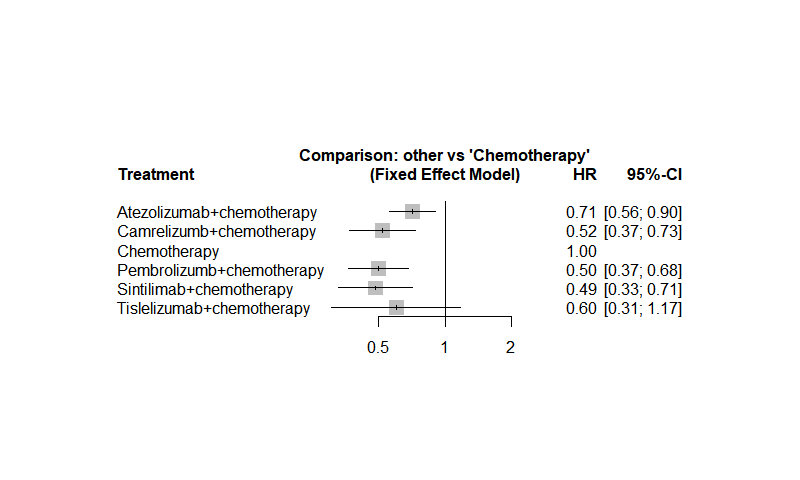 | 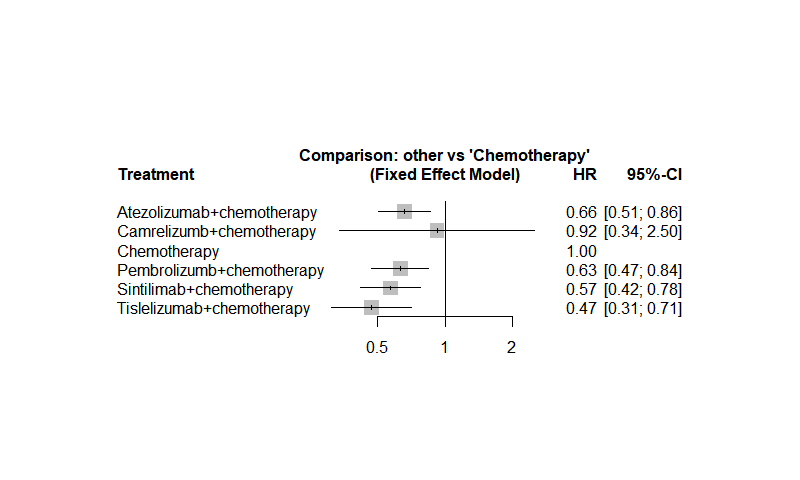 |
| Current or former smoker | Non-smoker |
| 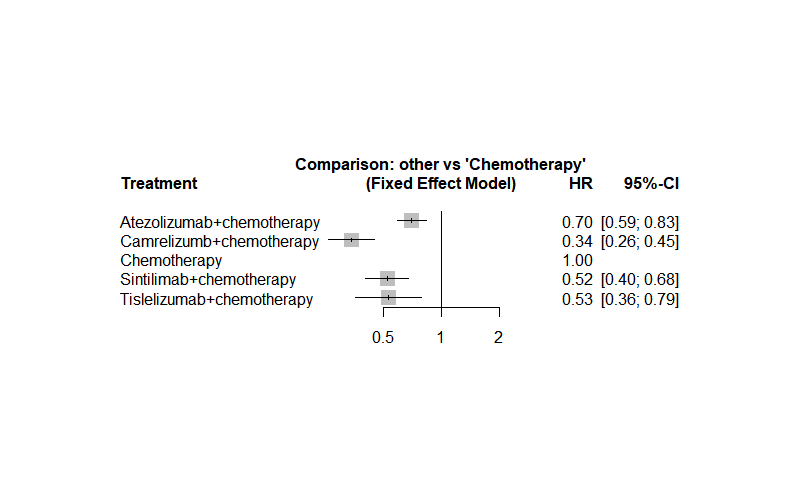 | 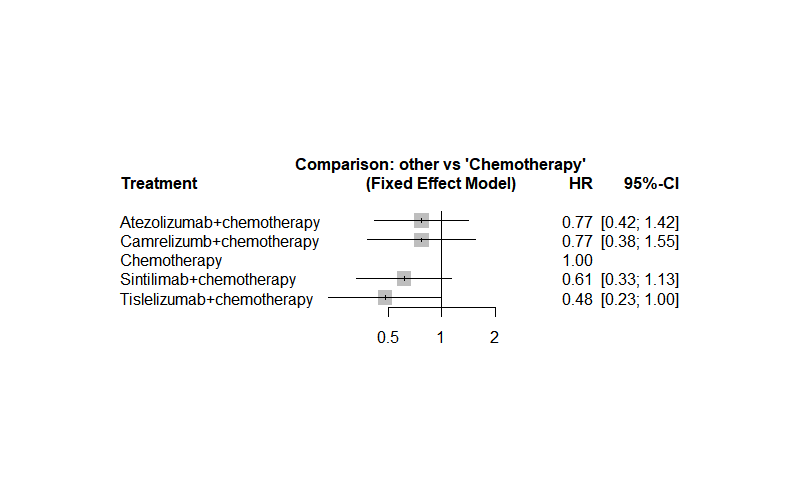 |
| ECOG: 0 | ECOG: 1 |
| 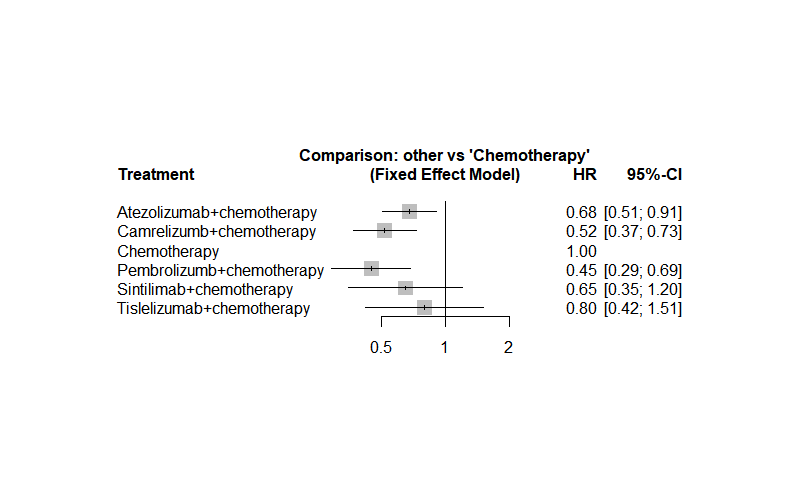 | 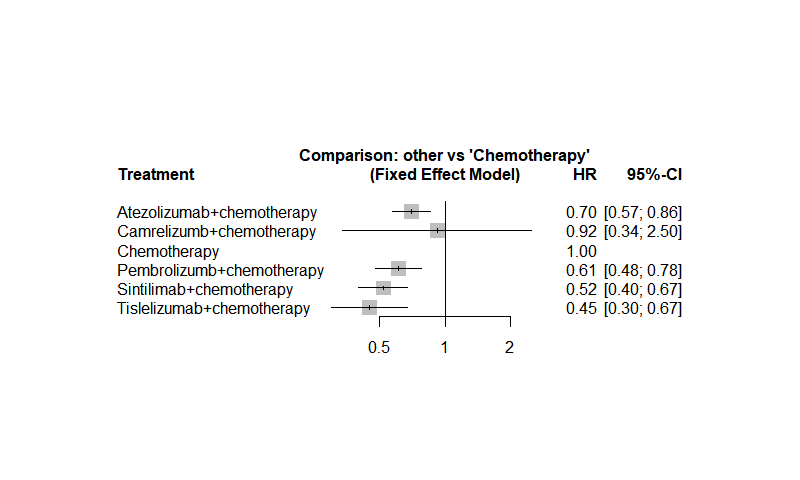 |
| ECOG indicates Eastern Cooperative Oncology Group; HR, hazard ratio; PD-L1, programmed cell death 1 ligand 1. | |
